# Novel non-phosphorylative pathway of pentose metabolism from bacteria

**DOI:** 10.1038/s41598-018-36774-6

**Published:** 2019-01-17

**Authors:** Seiya Watanabe, Fumiyasu Fukumori, Hisashi Nishiwaki, Yasuhiro Sakurai, Kunihiko Tajima, Yasuo Watanabe

**Affiliations:** 1 Department of Bioscience, Graduate School of Agriculture, Ehime University, 3-5-7 Tarumi, Matsuyama, Ehime 790-8566 Japan; 2 Faculty of Agriculture, Ehime University, 3-5-7 Tarumi, Matsuyama, Ehime 790-8566 Japan; 3 Center for Marine Environmental Studies (CMES), Ehime University, 2-5 Bunkyo-cho, Matsuyama, Ehime 790-8577 Japan; 4 Faculty of Food and Nutritional Sciences, Toyo University, 1-1-1 Izumino, Itakura-machi, Ora-gun, Gunma, 374-0193 Japan; 5 Department of Bio-molecular Engineering, Graduate School of Science and Technology, Kyoto Institute of Technology, Matsugasaki, Sakyo-ku, Kyoto, 606-8585 Japan

## Abstract

Pentoses, including D-xylose, L-arabinose, and D-arabinose, are generally phosphorylated to D-xylulose 5-phosphate in bacteria and fungi. However, in non-phosphorylative pathways analogous to the Entner-Dodoroff pathway in bacteria and archaea, such pentoses can be converted to pyruvate and glycolaldehyde (Route I) or α-ketoglutarate (Route II) via a 2-keto-3-deoxypentonate (KDP) intermediate. Putative gene clusters related to these metabolic pathways were identified on the genome of *Herbaspirillum huttiense* IAM 15032 using a bioinformatic analysis. The biochemical characterization of C785_RS13685, one of the components encoded to D-arabinonate dehydratase, differed from the known acid-sugar dehydratases. The biochemical characterization of the remaining components and a genetic expression analysis revealed that D- and L-KDP were converted not only to α-ketoglutarate, but also pyruvate and glycolate through the participation of dehydrogenase and hydrolase (Route III). Further analyses revealed that the Route II pathway of D-arabinose metabolism was not evolutionally related to the analogous pathway from archaea.

## Introduction

**Figure 1:**
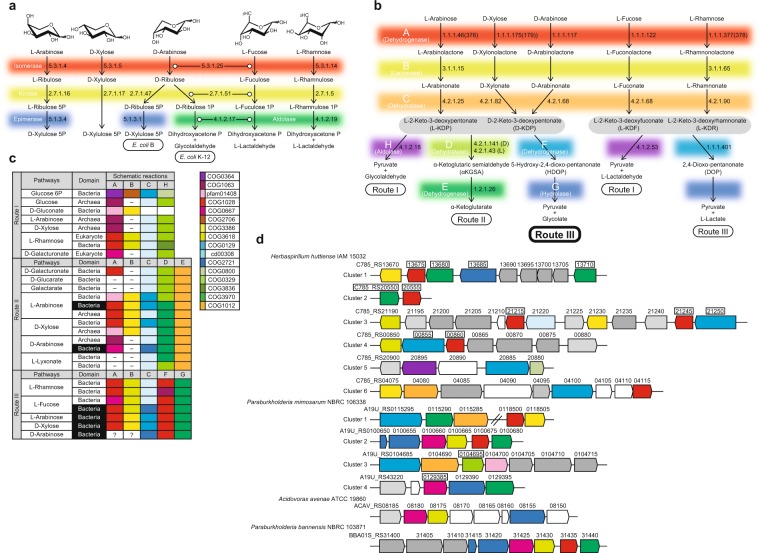
Pentose metabolism by microorganisms. (**a**) Schematic representation of phosphorylative pathways from bacteria. In the metabolism of D-arabinose, *E. coli* K-12 uses the same enzymes as the L-fucose pathway^44^, whereas *E. coli* strain B utilizes some of the enzymes involved in the D-ribitol pathway^45^. (**b**) Schematic representation of non-phosphorylative pathways analogous to the ED pathway. Pentose (and/or deoxyhexose) is commonly converted into 2-keto-3-deoxyacid-sugar, the subsequent metabolic fate of which is aldol-cleavage (Route I), dehydration (Route II), or dehydrogenation (Route III, in this study). (**c**) Comparisons of metabolic enzymes involved in non-phosphorylative pathways. Homologous genes are indicated in the same color and correspond to (**d**). White letters in boxes were discovered in the present study. (**d**) Schematic gene clusters related to non-phosphorylative sugar pathway(s). Putative genes in the box were purified and characterized in the present study. Genes in light- and deep-gray are putative transcriptional regulators and sugar transporters, respectively.

The breakdown of D-glucose is central for energy and biosynthetic metabolism throughout all domains of life. The most common glycolytic routes in bacteria are the Embden-Meyerhof-Parnas, the Entner-Doudoroff (ED), and the oxidative pentose phosphate pathways. The distinguishing difference between the two former glycolytic pathways lies in the nature of the 6-carbon metabolic intermediates that serve as substrates for aldol cleavage. For the ED pathway, this intermediate is D-2-keto-3-deoxy-6-phosphogluconate, from which pyruvate and D-glyceraldehyde 3-phosphate are formed, whereas for Embden-Meyerhof-Parnas pathway, D-glyceraldehyde 3-phosphate and dihydroxyacetone phosphate are produced from fructose-l,6-bisphosphate^1^. Alternatively, several hyperthermophilic and/halophilic archaea use a non-phosphorylative ED pathway that finally yields pyruvate and D-glyceraldehyde^2^. Schematic sugar conversion is almost analogous to that of the ED pathway, while the equivalent metabolic enzymes possess no evolutionary relationship^3–5^ (see Fig. 1c). However, pentoses, including D-xylose, L-arabinose, and D-arabinose, are metabolized through three main routes, which differs from D-glucose described above. The first pathway is present in bacteria, and uses isomerases (EC 5.3.1.-), kinases (EC 2.7.1.-), and epimerases (EC 5.1.3.-) to yield D-xylulose 5-phosphate (Fig. 1a). In the second pathway, mainly found in yeast and fungi, pentoses are commonly converted into D-xylulose 5-phosphate by reductases, dehydrogenases instead of isomerases, and epimerases^6^.

The third pathway for pentoses is (partially) analogous to the non-phosphorylative ED pathway, and has been classified into Routes I and II, in which pentoses are commonly converted into 2-keto-3-deoxypentonate (KDP) through the participation of aldose 1-dehydrogenase (EC 1.1.1.-), lactone-sugar hydrolase (lactonase) (EC 3.1.1.-), and acid-sugar dehydratase (EC 4.2.1.-) (schematic reactions A, B, and C in Fig. 1b). The “Route I” pathway of D-xylose and L-arabinose from bacteria and archaea^7–9^ is completely homologous to the non-phosphorylative ED pathway, and the KDP intermediate is cleaved through an aldolase reaction to pyruvate and glycolaldehyde (schematic reaction H). The analogous pathway is also found in the metabolism of deoxyhexoses, such as L-rhamnose and L-fucose, by bacteria, fungi, and/or archaea^10,11^. In the “Route II” pathway of D-xylose^12–14^, L-arabinose^15–18^, and D-arabinose^19–21^ from bacteria and/or archaea, the KDP intermediate is alternatively converted into α-ketoglutarate via α-ketoglutaric semialdehyde (αKGSA) by KDP dehydratase (EC 4.2.1.141)^22^ and αKGSA dehydrogenase (EC 1.2.1.26) (schematic reactions D and E). αKGSA is also a metabolic intermediate involved in D-galacturonic acid and hexaric acids (D-glucarate and D-galactarate) pathways from bacteria^23–25^. On the other hand, in the modified non-phosphorylative pathway of L-rhamnose and L-fucose from bacteria and/or archaea^26–28^, each L-2-keto-3-deoxyrhamnonate (L-KDR) and L-2-keto-3-deoxyfuconate intermediate (L-KDF) is converted into pyruvate and lactate via (putative) 2,4-dioxo-pentanonate by the sequential actions of dehydrogenase and hydrolase (schematic reactions F and G, the “Route III” pathway). Metabolic genes related to these non-phosphorylative pentose (and deoxyhexose) pathways often cluster together with the putative sugar (ABC-type) transporter genes and transcriptional regulator gene on the genomes of bacteria and archaea. Furthermore, the numbers of protein superfamilies belonging to these metabolic enzymes are limited, and there is significant phylogenetic mosaicism between them (Fig. 1c)^10^.

Based on these findings, we herein focused on the putative gene cluster(s) of *Herbaspirillum huttiense* IAM 15032 related to the Route II pathway of pentose metabolism. The biochemical characterization of C785_RS13685, one of the components encoded to D-arabinonate dehydratase, differed from the known acid-sugar dehydratases. The biochemical characterization of the remaining components and genetic expression analysis revealed that D- and L-KDP intermediates, produced from D-xylose, L-arabinose, and D-arabinose, were converted into not only α-ketoglutarate, but also pyruvate and glycolate via a 5-hydroxy-2,4-dioxo-pentanonate intermediate. To the best of our knowledge, this is the first study to show that pentoses are also metabolized through the non-phosphorylative Route III pathway, and that the non-phosphorylative D-arabinose pathway is operative not only in archaea^19–21^, but also in bacteria.

## Results and Discussion

### Putative gene clusters related to pentose metabolism from *H. huttiense* IAM 15032

A preliminary homology search was performed against bacterial genome sequences using metabolic genes involved in several sugar pathways analogous to the ED pathway as a probe. In the present study, we focused on *H. huttiense* IAM 15032. There are at least six interesting gene clusters on the genome of this bacterium (Fig. 1d and Supplementary Discussion). Among them, clusters 5 and 6 appear to be responsible for the metabolism of D-glucose 6-phosphate and sulfoquinovose (6-deoxy-6-sulfoglucose)^29^, respectively. Among cluster 1, the COG3970 (C785_RS13680 and C785_RS13710) and COG1028 (C785_RS13675) proteins (genes) are clustered with COG2721 (C785_RS13685), which is not contained in Fig. 1c. Since COG2721 consists of several dehydratases and/or hydrolases (see below), we postulated that the C785_RS13685 gene encodes a novel pentonate dehydratase, which differs from the known COG0129 and cd00308. In the present study, we were successful in homogeneously purifying all target proteins, including C785_RS13685, as (His)_6_-tagged enzymes (Fig. S1).

### Characterization of C785_RS13685 as a novel D-arabinonate dehydratase

**Figure 2:**
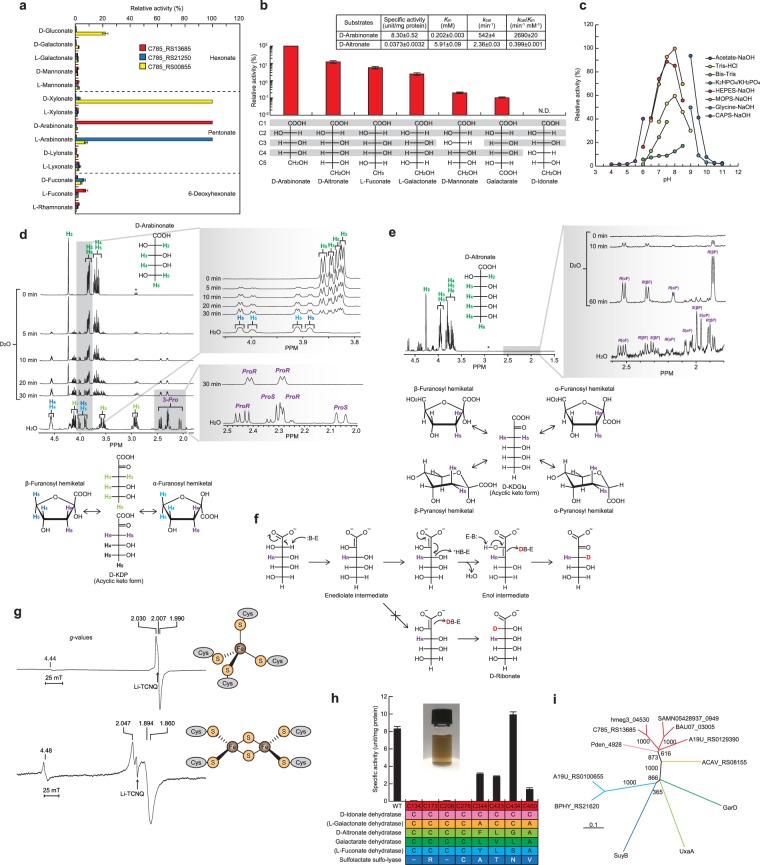
Functional characterization of C785_RS13685 as a D-arabinonate dehydratase. (**a**,**b**) Substrate specificity. The assay was performed by end-point (after 30 min) (**a**) and time course measurements (**b**) using the semicarbazide method. Relative values were expressed as percentages of the values obtained with D-arabinonate (means ± SD, *n* = 3). Below the bars, Fischer projections of the tested acid-sugar are shown. N.D. Not determined. *Inset*. Kinetic parameters, assessed by the spectrophotometric assay method using a coupling enzyme. (**c**) Effects of pH on activity. The indicated buffers were used instead of 50 mM HEPES-NaOH (pH 7.2) under standard assay conditions. (**d**) ^1^H NMR spectra of D-arabinonate and D-KDP, the dehydrated product of C785_RS13685 in D_2_O and H_2_O, showing the assignments of all protons. Asterisks are peaks derived from an internal standard. The lower panel indicates the structures of the acyclic keto and cyclic furanose forms of the D-KDP product. (**e**) ^1^H NMR spectrum of D-altronate showing the assignments of all protons, and the partial ^1^H NMR spectrum of the product obtained by a reaction in D_2_O and H_2_O showing resonances associated with 3-*ProR* and 3-*ProS*. The lower panel indicates the structures of the acyclic keto and cyclic furanose and pyranose forms of the D-2-keto-3-deoxygluconate product. (**f**) Proposed catalytic mechanism by C785_RS13685 in D_2_O. No partition of the enediolate intermediate to the 2-epimerization reaction is observed, in contrast to L-fuconate dehydratase of the enolase type^28^. (**g**) X-band EPR spectra of DTT-reduced C785_RS13685 at 8 K (upper panel) and Na_2_S_2_O_4_-reduced C785_RS21250 at 20 K (lower panel). (**h**) Effects of the substitution of cysteine to serine on activity. Values are the means ± SD, *n* = 3. The inset photograph is the purified recombinant enzyme (~40 mg/ml) of the wild-type. The bottom figure indicates the corresponding amino acid residues of the other UxaA/GarD superfamily members to the site-directed mutated sites of C785_RS13685. Functions in parentheses were assumed based on the gene context. The color corresponds to (**i**). Multiple amino acid sequence alignments are shown in Fig. S3. (**i**) Phylogenetic tree of the UxaA/GarD superfamily including L-arabinonate dehydratase (this study), consisting of nine subfamilies. The number on each branch indicates the bootstrap value.

In order to estimate the enzyme function of the C785_RS13685 gene (protein), fourteen acid-sugars, including pentonates, chemically synthesized from the corresponding sugar were tested as substrates “without additives”; all known acid-sugar dehydratases were Mg^2+^-dependent enzymes. In semicarbazide end-point measurements (see “Methods”), only D-arabinonate and L-fuconate were identified as active substrates (Fig. 2a). We then prepared D-altronate and L-galactonate with the same C2, C3, and C4 configurations as D-arabinonate (Fig. 2b). The results of the time course analysis showed that C785_RS13685 utilized D-arabinonate (100%), D-altronate (12%), L-fuconate (5.6%), and L-galactonate (2.4%). pH dependence was estimated using the same (end-point) method: an optimum pH of 7.0–8.0 (Fig. 2c). The dehydration product from D-arabinonate by C785_RS13685 was purified by anion-exchange chromatography, and the ^1^H NMR spectrum was identical to a mixture of the acyclic keto and α-/β-furanosyl hemiketal forms of D-KDP; this interconversion was reported previously^30^ (Fig. 2d). Furthermore, a kinetic analysis using a coupling enzyme (see “Methods”) showed that the *k*_cat_/*K*_m_ value with D-arabinonate (2,690 min^−1^·mM^−1^) was at the physiological level, and ~6,700-fold higher than that that with D-altronate (0.399 min^−1^·mM^−1^) (inset of Fig. 2b). Three acid-sugar dehydratases that utilize D-arabinonate as the substrate have been identified, but belong to the enolase superfamily; D-arabinonate dehydratase from Archaeon *Sulfolobus solfataricus*^19^; L-fuconate dehydratases from *Xanthomonas campestris* and humans^28,31^. Collectively, these results suggest that the C785_RS13685 protein is a novel D-arabinonate dehydratase enzyme, and that strict substrate specificity may be involved in (unidentified) the physiological metabolic pathway (of D-arabinose, see below).

### Catalytic mechanism of D-arabinonate dehydratase

The stereochemical course of the dehydration of D-arabinonate catalyzed by C785_RS13685 was assessed by comparing the ^1^H NMR spectra of the products obtained in D_2_O and H_2_O; one prochiral hydrogen of the C3 of each α-/β-hemiketal anomer was stereospecifically deuteriated in the 3-*ProS* position to yield 2-keto-3-deoxy-[3(*S*)-^2^H]-D-pentonate (Fig. 2d). Although acid-sugar library screening showed low-level activity with D-altronate (and L-fuconate), the ^1^H NMR spectrum of the product formed after the incubation (with an ~100-fold larger amount of the enzyme than D-arabinonate) was not that expected for dehydration initiated by the abstraction of an α-proton to the carboxylate group (Fig. S2). On the other hand, the product from D-altronate was identified as a mixture of four species, the α-/β-anomeric pairs of the furanosyl and pyranosyl hemiacetals of D-2-keto-3-deoxygluconate, and the incorporation of solvent-derived deuterium in the 3-*ProS* position was also observed (Fig. 2e). These results suggested that the catalytic mechanism consists of^1^ the abstraction of the α-proton of the substrate by a polyprotic active site base to generate an enediolate intermediate^2^, vinylogous β-elimination to accomplish dehydration of the enediolate intermediate to yield the enol intermediate, and^3^ replacement of the departing 3-OH group with solvent deuterium with the inversion of its configuration (Fig. 2f). When D-arabinonate was used with L-fuconate dehydratase (enolase type) as the substrate, the “Mg^2+^-stabilized” enediolate intermediate partitioned between similar dehydration and 2-epimerization reactions (to yield D-ribonate) (Fig. 2f)^28^.

Purified C785_RS13685 was brown in color (see inset of Fig. 2h), and stored under buffer containing 0.1 mM Fe^2+^ and 10 mM DTT for several months. Therefore, in order to examine the nature of the oxidized form with ferric ion in more detail, an electron paramagnetic resonance (EPR) analysis recorded for the frozen solution was performed (Fig. 2g). Under aerobic conditions, an intense signal centered at *g* ≈ 2 with a spectral width ca. 15 mT (no temperature sensitive), and anisotropic EPR signals at *g* = 4.41 were observed. The latter species is originate from ferric ion, taking the ferric high-spin state (*S* = 5/2) such as a rubredoxin (*g* = 4.3, <20 K), supporting that the frozen solution contained oxidized form of the protein with ferric ion. While the *g*-values of the former species (*g*_1_ = 2.0323, *g*_2_ = 2.0025, and *g*_3_ = 2.0011) is comparable with those of such as [3Fe-4S]^+^ ferredoxin (*g*_1_ = 2.02, *g*_2_ = 2.00, and *g*_3_ = 1.97, <20 K), their “reduced” forms should be EPR-silent, in contrast of C785_RS13685. There is one report of EPR analysis of D-altronate dehydratase (UxaA), one member of the same protein superfamily as C785_RS13685 (see next section)^32^. Upon addition of substrate under aerobic condition, the major signal (*g* ≈ 2) disappears and a dramatic rise of the EPR signals of ferric high-spin species (*g* = 4.15) is observed, similar to “oxidized” rubredoxin, by which it is concluded that this enzyme is the same type. Collectively, although further analysis should be necessary, it is no doubt that C785_RS13685 is completely different from pentonate dehydratases of the ILVD/EDD type such as C785_RS21250 (see below); in fact, the *g*-values (*g*_1_ = 2.047, *g*_2_ = 1.894, and *g*_3_ = 1.860, <40 K) showed reasonable accordance with [2Fe-2S]^−^ protein (Fig. 2g)^33–35^.

### Phylogenetic analysis of D-arabinonate dehydratase

As expected from the preliminary annotation, C785_RS13685 belongs to the D-altronate dehydratase (UxaA)/D-galacarate dehydratase (GarD) superfamily (COG2721)^32^, in which four functional enzymes are contained: the archetypical UxaA (EC 4.2.1.7) and GarD (EC 4.2.1.42), D-idonate dehydratase (EC 4.2.1.-), and sulfolactate sulfo-lyase (SuyAB, EC 4.4.1.24); only the latter is a heterometric structure (Fig. S3). Regarding the physiological roles of bacteria, the two former enzymes produce 2-keto-3-deoxygluconate in uronic acid metabolism^36^, while the two latter enzymes are involved in L-glucose and L-cysteate (2-amino-3-sulfopropionate) metabolism, respectively^37,38^. Among these members, C785_RS13685 was closely related to D-idonate dehydratase (from *Paracoccus denitrificans* PD1222, 67% identity), which catalyzes the dehydration of D-idonate to D-2-keto-3-deoxygalactonate (Fig. 2i)^37^. This was unexpected because D-idonate shares the same configuration with D-arabinonate at C2 and C3 only (Fig. 2b). Furthermore, comparisons with gene contexts indicated that the A19U_RS0100650 and A19U_RS0100655 genes from *Paraburkholderia mimosarum* NBRC 106338 encoded L-fuconate dehydratase with a heteromeric structure, and also that ACAV_RS08155 from *Acidovorax avenae* ATCC 19860 functioned as a L-galactonate dehydratase involved in (unidentified) L-galactose metabolism (unpublished data) (Fig. 1d). As described above, if C785_RS13685 is a rubredoxin-type protein, one ferric iron is coordinated to four cysteinyl sulfurs. The amino acid sequence of C785_RS13685 contained eight cysteine residues, among which only Cys134, Cys173, Cys208, and Cys276 were conserved in these UxaA/GarD superfamily enzymes, except for sulfolactate sulfo-lyase (Figs 2h and S3), conforming with their common ferric iron-binding sites, as described above.

### Functional characterization of C785_RS13680 and C785_RS13710

**Figure 3:**
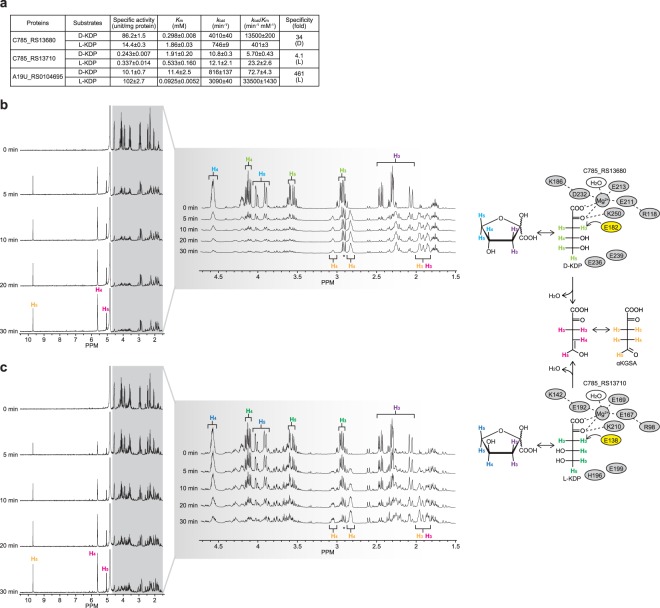
Functional characterization of C785_RS13680 and C785_RS13710 as KDP dehydratase. (**a**) Kinetic parameters, assessed by the spectrophotometric assay method using a coupling enzyme. Values are the means ± SD, *n* = 3. Dehydration of D-KDP and L-KDP by C785_RS13680 (**b**) and C785_RS13710 (**c**), respectively, in D_2_O, monitored by ^1^H NMR spectroscopy. The putative product, αKGSA, was identified as a mixture with its enol form. Light panels also contain schematic diagrams showing the predicted active sites. Both enzymes possessed not only several amino acid residues commonly conserved in the FAH/MhpD superfamily, but also specific glutamate residues for their catalysis (yellow) (see Fig. S4).

C785_RS13680 and C785_RS13710 both belonged to the fumarylacetoacetate hydrolase (FAH) superfamily (COG3970) and 2-hydroxyhepta-2,4-diene-1,7-dioate isomerase/5-carboxymethyl-2-oxohex-3-ene-1,7-dioate decarboxylase (MhpD) superfamily (COG0179) (Fig. S4). Although a poor phylogenetic relationship is known to exist between the members of this superfamily (in particular, the latter), the two proteins may possess the specific glutamate residue for archaeal D-KDP dehydratase, which initially abstracts the C3 proton of the substrate; Glu182 and Glu138, respectively^19,21^. C785_RS13680 and C785_RS13710 utilized not only D-, but also L-KDP as the substrate (10 mM). The *k*_cat_/*K*_m_ value with D-KDP of C785_RS13680 was 34-fold higher than that with L-KDP, whereas C785_RS13710 showed moderate stereochemical specificity for L-KDP (Fig. 3a). Each reaction product from D- and L-KDP by C785_RS13680 and C785_RS13710 was identified as αKGSA by a ^1^H NMR analysis (Fig. 3b,c); a mixture with its enol form, as reported previously^39^. Collectively, these results strongly suggest that C785_RS13680 and C785_RS13710 catalyze the dehydration reaction of D- and/or L-KDP with a poor phylogenetic relationship to the archaeal enzyme (Fig. S4b).

L-KDP dehydratase is considered to belong to the dihydrodipicolinate synthase/*N*-acetylneuraminate lyase superfamily (COG0329), the catalytic mechanism of which involves the formation of a Schiff base between the substrate and active site lysine residue^17^; this is the first study on the FAH/MhpD-type enzyme. We herein found that A19U_RS0104695 from *P. mimosarum* NBRC 106338, a typical homolog (Fig. 1d), showed strict specificity for L-KDP (Fig. 3a), conforming with an involvement in L-arabinose metabolism only. On the other hand, Saci_1939 from the Archaeon *S. acidocaldarius* may function as a bifunctional dehydratase for D- and L-KDP in the metabolism of D-xylose and L-arabinose^8,9^.

### Functional characterization of C785_RS13675, C785_RS20555, and C785_RS20550

**Figure 4:**
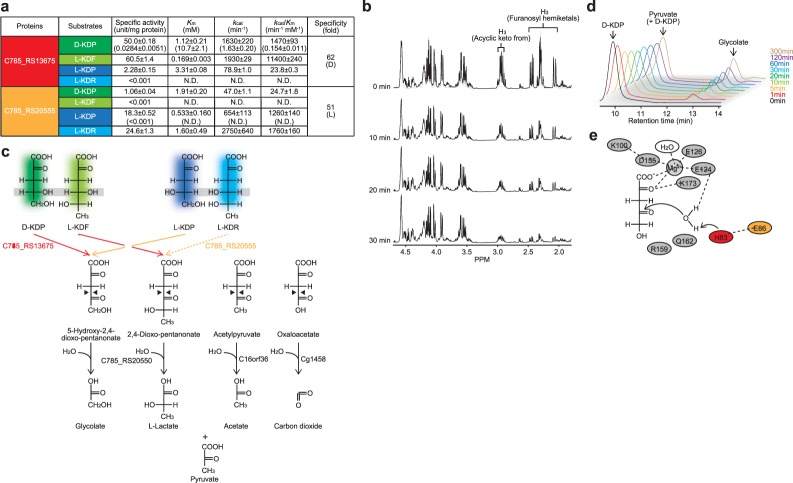
Alternative metabolic fate of KDP. (**a**) Kinetic parameters using NAD^+^ as a coenzyme of C785_RS13675 and C785_RS20555. Values are the means ± SD, *n* = 3. Values in parentheses are NADP^+^-dependent activities. (**b**) Dehydrogenation of D-KDP by C785_RS13675 in D_2_O, monitored by ^1^H NMR spectroscopy. Only 3 protons derived from the acyclic keto and furanosyl hemiketal forms of D-KDP are shown. Gray shadow indicates the spectrum of NAD^+^. (**c,d**) Schematic of enzyme reaction products from D-KDR and L-KDF, and L-KDP and L-KDR formed by C785_RS13675 and C785_RS20555, respectively, together with C785_RS20550. A HPLC analysis of the former is shown in (**d**). L-KDR did not appear to be a physiological substrate for C785_RS13710 (bashed line) (see in text). C16orf36 and Cg1458 are phylogenetically related to C785_RS20550 (Fig. S4), and their reactions commonly involve the cleavage of C–C bonds (black triangles) to yield pyruvate, one of the products. (**e**) Schematic diagrams showing the predicted active sites of C785_RS20550, similar to Fig. 3b,c. Histidine (red) and glutamate residues (orange) are specifically important for catalysis.

C785_RS13675, C785_RS20555, and C785_RS20550 are physiologically related to L-KDF 4-dehydrogenase (XCC4067), L-KDR 4-dehydrogenase (SKA58_03590), and 2,4-dioxo-pentanonate hydrolase (SKA58_03585), which are involved in the non-phosphorylative Route III pathways of L-fucose and/or L-rhamnose metabolism of bacteria; sequential identities of 58%, 51%, and 56%, respectively^26,28^ (Figs S4 and S5). Among them, zymogram staining and a kinetic analysis revealed that C785_RS13675 and C785_RS20555, belonging to the short-chain dehydrogenase/reductase (SDR) superfamily (COG1028), function as a NAD^+^-dependent D-KDP/L-KDF and L-KDP/L-KDR (4-)dehydrogenase, respectively, and their coenzyme specificities and stereoselectivities of substrates were very strict (Fig. S6a and Fig. 4a). Since the non-phosphorylative L-rhamnose pathway^9^ may not be operative in *H. huttiense* IAM 15032 (data not shown), the physiological substrate for C785_RS20555 is only L-KDP (see Fig. 4c). Although a ^1^H NMR analysis showed that as a result of abstraction of the proton on the C4 of D-KDP by C785_RS13675 in the presence of NAD^+^, resonance associated with the protons on C3 rapidly decreased with time (Fig. 4b), it was impossible to directly identify the putative reaction product (5-hydroxy-2,4-dioxo-pentanonate). Alternatively, in the HPLC analysis, when D-KDP was incubated with C785_RS13675 and C785_RS20550 in the presence of NAD^+^, a novel peak with a later retention time (~13 min) appeared and was identical to glycolate (Fig. 4d). When L-KDF was used instead of D-KDP, the product was lactate (data not shown). Among several FAH/MhpD members, C785_RS20550 was related to acylpyruvate hydrolase (C16orf36) and fumarylacetoacetate hydrolase (Cg1458)^40,41^ (Fig. S4). These enzymes catalyze similar cleavage reactions of the C–C bond of the substrate (Fig. 4c), and possess two specific histidine and glutamate residues, which form the catalytic triad with water; His83 and Glu86 in C785_RS20550 (Fig. 4e). Collectively, these results suggest that D- and/or L-KDP is metabolized through a homologous route with Route III of the non-phosphorylative deoxyhexose pathway (Fig. 1b).

### Characterization of other metabolic genes related to pentose pathways

Clusters 3 and 4 of *H. huttiense* IAM 15032 (Fig. 1d) may consist of genes (enzymes) related to schematic reactions A, B, and C only (see Fig. 6a). Among them, acid-sugar library screening and the kinetic analysis revealed that C785_RS21250 and C785_RS00855, belonging to the ILVD/EDD superfamily (COG0129), functioned as dehydratases for L-arabinonate and D-xylonate with high substrate specificities, respectively (Figs 2a and S7), conforming with their phylogenetic analysis (Supplementary Discussion). On the other hand, C785_RS21220 is sequentially similar to L-fuconate dehydratase of the enolase type (from *X. campestris*, identity of 57%)^28^.

**Figure 5:**
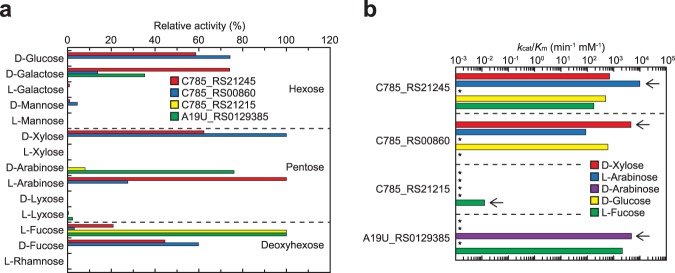
Functional characterization of C785_RS21215, C785_RS21245, C785_RS00860, and A19U_RS0129385 as aldose 1-dehydrogenases. (**a**) Substrate specificity. Relative values were expressed as percentages of their maximum values (means ± SD, *n* = 3). (**b**) Comparisons of *k*_cat_/*K*_m_ values for D-xylose, L-arabinose, D-arabinose, D-glucose, and L-fucose, taken from the values in Table S2 (means ± SD, *n* = 3). Arrows indicate each physiological substrate.

Bacteria do not generally utilize aldose and the corresponding acid-sugar as a sole carbon source; for example, *Pseudomonas aeruginosa* PAO1 only grew on L-lyxonate, and not on L-lyxose (Fig. 1c)^42^. Since *H. huttiense* IAM 15032 grew on D-xylose, L-arabinose, D-arabinose, or L-fucose (see next section), we postulated that the three SDR superfamily proteins, C785_RS21215, C785_RS21245, and C785_RS00860 (Fig. 1d), catalyze the NAD(P)^+^-dependent oxidization of these aldoses to the corresponding lactone sugars (Supplementary Discussion). Library screening of fourteen aldoses initially showed that NADP^+^-dependent C785_RS21215 was strictly specific for L-fucose. Although NAD^+^-dependent C785_RS21245 and C785_RS00860 showed relative broad substrate specificities (Fig. 5a), the *k*_cat_/*K*_m_ value for L-arabinose of the former (9,720 min^−1^·mM^−1^) was 2.9-, 14-, and 20-fold higher than those of D-galactose, D-xylose, and D-glucose, respectively, whereas the *k*_cat_/*K*_m_ value for D-xylose of the latter (4,530 min^−1^·mM^−1^) was 7.5-, 13-, and 52-fold higher than those of D-glucose, D-fucose, and L-arabinose, respectively (Fig. 5b and Table S1). These substrate specificities were also observed in the in-gel assay (zymogram staining; Fig. S6b).

Collectively, these results strongly suggest that cluster 3 is responsible for the metabolism of L-arabinose and L-fucose, whereas cluster 4 is for D-xylose. Furthermore, a phylogenetic analysis revealed that C785_RS21215 and C785_RS21245 are novel types of L-fucose 1-dehydrogenase and L-arabinose 1-dehydrogenase from bacteria, respectively, differing from the known enzymes, indicating their convergent evolution (Supplementary Discussion).

### Gene expression analysis of *H. huttiense* IAM 15032

**Figure 6:**
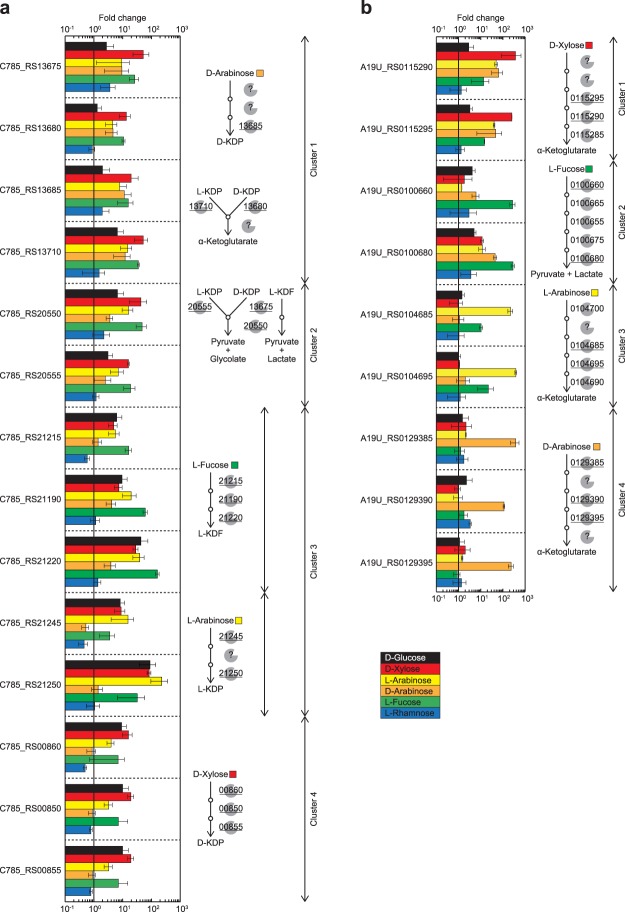
Transcriptional analysis by qRT-PCR of *H. huttiense* IAM 15032 (**a**) and *P. mimosarum* NBRC 106338 (**b**). The strains were cultivated overnight on nutrient medium and replaced with minimal medium containing 1% (w/v) of the indicated sugar (D-glucose, D-xylose, L-arabinose, D-arabinose, L-fucose, or L-rhamnose) for 4 h. Assays were performed in triplicate, and the data are averages and standard deviations of the results. The average ratio is the fold difference compared to cells grown without sugar. The right panel indicates the schematic representation of sugar pathways related to gene cluster, based on biochemical characterization. The underlined genes were analyzed by qRT-PCR.

We identified minimal medium for the growth of *H. huttiense* IAM 15032 (and *P. mimosarum* NBRC 106338, see below), as described in “Methods”. In order to estimate the potential metabolism of sugar(s), a quantitative real-time PCR (qRT-PCR) analysis was performed using cells grown on D-glucose, D-xylose, L-arabinose, D-arabinose, L-fucose, and L-rhamnose as a sole carbon source (Fig. 6a). Despite the lack of involvement in hexose metabolism based on biochemical data, D-glucose significantly up-regulated the transcription of clusters 1~4, whereas L-rhamnose did not. Similar phenomena have been reported in the extremely thermophilic bacterium *Caldicellulosiruptor saccharolyticus* DSM 8903, in which no catabolite repression by D-glucose on the use of D-xylose was noted^22,43^. In the case of *H. huttiense* IAM 15032, since D-glucose and D-gluconate were additional substrates for C785_RS00860 and C785_RS00855 (Figs 5 and S7), respectively, the up-regulation of these genes by D-glucose may partially metabolize its sugar through non-phosphorylative intermediates, in addition to the ED pathway, similar to Archaeon *S. solfataricus* and *S. acidocaldarius*; in their non-phosphorylative Route I pathway of D-glucose, D-xylose and L-arabinose, only dehydratases for D-gluconate and D-xylonate/D-arabinonate are two separate enzymes^8,9^.

In comparison with D-glucose, C785_RS00855 (2.0-fold) and C785_RS00860 (2.1) (for D-xylose → D-KDP), C785_RS21250 (2.6) and C785_RS21245 (2.3) (for L-arabinose → L-KDP), and C785_RS21215 (3.6) and C785_RS21220 (3.8) (for L-fucose → L-KDF) genes were induced by D-xylose, L-arabinose, and L-fucose, respectively, conforming with the biochemical characterization of the metabolic genes. On the other hand, D-xylose, L-arabinose, “D-arabinose”, and L-fucose up-regulated the transcription of clusters 1 and 2, in which metabolic genes for D- and D-KDP are contained. Collectively, these results strongly suggested that these pentoses entered into Routes II and III of the non-phosphorylative pathway.

### Identification of metabolic genes involved in the non-phosphorylative D-arabinose pathway from bacteria

In *E. coli* K-12, regulatory mutations in the L-fucose pathway, consisting of isomerase, kinase, and aldolase, lead to growth on D-arabinose (Fig. 1a)^44^. However, *E. coli* strain B metabolizes D-arabinose via D-ribulose 5-phosphate, involved in the so-called “D-ribitol pathway” without genetic mutations^45^. Although *H. huttiense* IAM 15032 may metabolize D-arabinose via the non-phosphorylative pathway instead of these known pathway(s), there is no candidate of D-arabinose 1-dehydrogenase (gene). On the other hand, the gene contexts of clusters 1, 2, and 3 from *P. mimosarum* NBRC 106338 (Fig. 1d) were typical for the D-xylose (Route II), L-fucose (Route III) and L-arabinose pathways (Route II), respectively, the gene expression of which was clearly reasonable for the metabolism of each sugar, relative to that from *H. huttiense* IAM 15032 (Fig. 6b). In cluster 4, the D-arabinonate dehydratase (A19U_RS0129390) and D-KDP dehydratase (A19U_RS0129395) genes were clustered with the A19U_RS0129385 gene, a member of the aldo/keto reductase superfamily (COG0667), and their expression was specifically induced by D-arabinose. Biochemical characterization using the recombinant protein (Fig. S1) revealed that among the fourteen aldoses tested, D-arabinose and L-fucose (and L-galactose) were substrates for NAD^+^-dependent dehydrogenation and each *k*_cat_/*K*_m_ value was similar (4,700 and 2,130 min^−1^·mM^−1^) (Fig. 5a,b and Table S1); similar dual specificity was found in known L-fucose 1-dehydrogenase^46,47^. These results strongly suggest the involvement of cluster 4 in the non-phosphorylative Route II pathway of D-arabinose metabolism. On the other hand, the D-arabinose-negative phenotype of *E. coli* wasn’t compensated by introduction of the metabolic genes, related to D-arabinose pathway(s) (Route II or III) (Supplementary Discussion and Fig. S8). Indeed, although western blot analysis using (His)_6_-tag attached to the N-terminus of all proteins revealed their functional expressions in *E. coli* cells, expression level of the several genes (in particular, A19U_RS0129385) is extremely low. Further experiment should be necessary to estimate the physiological meaning of the pathway of D-arabinose metabolism *in vivo*.

In the equivalent Route II pathway from *S. solfataricus*^19,20^, D-arabinose 1-dehydrogenase (SSO1300) and D-arabinonate dehydratase (SSO3124) belong to the medium-chain dehydrogenase/reductase (COG1063) and enolase superfamilies (cd00308), respectively, which differ from bacteria (Fig. 1c), indicating their convergent evolution. The archaeal D-arabinose pathway “partially” overlaps with the Route I pathway of L-fucose metabolism, in which SSO1300 and SSO3214 function as L-fucose 1-dehydrogenase and D-fuconate dehydratase, respectively^11^. On the other hand, gene clusters for D-arabinose and L-fucose metabolism by *P. mimosarum* NBRC 106338 (clusters 4 and 2, respectively, Fig. 1d) were only induced by each sugar (Fig. 6b); there was no metabolic promiscuity between D-arabinose and L-fucose.

In cases of Route II of the non-phosphorylative pathway and *E. coli* strain B pathway, the major portion of the total energy is gained via the TCA cycle and the oxidative pentose phosphate pathways, respectively. When compared other D-arabinose degradation pathways to make pyruvate in terms of NAD(P)H and ATP, D-arabinose degradation by *E. coli* K-12 would yield 3 NAD(P)H and 1 ATP per molecule D-arabinose, as the formed dihydroxyacetone phosphate is utilized via glyceraldehyde-3-phosphate in the Embden-Meyherhof-Parnas pathway (Fig. S9). On the other hand, both Routes I and III of the non-phosphorylative pathway would produce only 3 NAD(P)H per molecule D-arabinose and are, therefore, energetically comparable. The later Route allows to convert D-arabinose to the same pyruvate and glycolate products without involvement of the physiologically toxic aldehyde (glycolaldehyde), which may be one of the potential benefits.

## Conclusion

The gene context in the bacterial genome facilitates estimations of potential substrates, by which we may register D-arabinonate dehydratase, involved in the novel D-arabinose pathway from bacteria, in the UxaA/GarD superfamily, and discover an alternative metabolic fate for KDP intermediate(s) that differ from dehydration and aldol-cleavage. Enhancements in the “metabolic gene catalog (Fig. 1c)” may contribute to the identification of other non-phosphorylative sugar pathways from microorganisms.

## Methods

### Plasmid construction for the expression of recombinant proteins

The PCR amplification was performed by using primers shown in Table S2 and *H. huttiense* NBRC 102521 or *P. mimosarum* NBRC 106338 genomic DNA as a template. The amplified product was digested with BamHI and HindIII and then inserted into pQE-80L (Qiagen), a plasmid vector for conferring an N-terminal (His)_6_-tag on the proteins expressed, in order to obtain each pQE-based expression plasmid. Regarding the expression of the C785_RS13710 gene in *P. putida* cells, a DNA fragment of the (His)_6_-C785_RS13710-*t*_0_ terminator was amplified by PCR using pQE/C785_RS13710 as a template, and introduced into the SalI-EcoRI sites in pUCP26KmAhpC_p_ ^48^ in order to obtain pUCP/C785_RS13710. A site-directed mutation was introduced into the C785_RS13685 gene by sequential steps of PCR using sense and antisense primers (Table S2) and pQE/C785_RS13685 as a template, in which each cysteine residue codon (TGC) was replaced with serine (AGC).

### Expression and purification of the recombinant protein

All pQE-based expression plasmids were transformed into *E. coli* strain DH5α cells for protein expression, whereas pUCP/C785_RS13710 was into *P. putida* KT2442-oxyR1^49^. Transformed *E. coli* cells was grown at 37 °C to a turbidity of 0.6 at 600 nm in LB medium containing ampicillin (50 mg/liter). After the addition of 1 mM isopropyl-β-D-thiogalactopyranoside (IPTG), the culture was grown for a further 6 h to induce the expression of the (His)_6_-tagged protein. On the other hand, *P. putida* KT2442-oxyR1^49^ harboring pUCP/C785_RS13710 was grown at 30 °C overnight in LB medium containing kanamycin (50 mg/liter). The harvested cells were suspended in in Buffer A (50 mM sodium phosphate buffer (pH 8.0) containing 300 mM NaCl and 10 mM imidazole). The suspension was lysed by sonication, and debris was cleared by centrifugation. The supernatant was applied to Ni-NTA spin column (Qiagen) equilibrated with Buffer A. After washing with Buffer B (50 mM sodium phosphate buffer (pH 8.0) containing 300 mM NaCl, 10% (v/v) glycerol, and 50 mM imidazole), the enzymes were eluted with Buffer C (pH 8.0, as Buffer B but containing 250 mM instead of 50 mM imidazole). The purified enzymes were concentrated by ultrafiltration, dialyzed against 50 mM Tris-HCl buffer (pH 8.0) containing 50% (v/v) glycerol, and stored at –35 °C until used. If necessary, 1 mM MgCl_2_ was employed as a buffer system. In the case of C785_RS13685, HEPES-NaOH buffer (pH 7.2) containing 0.1 mM (NH_4_)_2_Fe(SO_4_)_2_·6H_2_O, 10 mM DTT, and 50% (v/v) glycerol was used (see the text).

### Substrates

All acid-sugars were prepared by hypoiodite-in-methanol oxidization from the corresponding sugars as a K^+^ or Ba^2+^ salt^50^. The solution containing acid-sugar was then purified by using the column of an AG^®^ 1-X8 Resin (200–400 mesh, formate form) (Bio-Rad)^10^. Fractions containing acid-sugars were combined and lyophilized to yield the corresponding lactone-sugars. Acid-sugars were obtained by the base hydrolysis of the lactone-sugar, according to the method of Yew *et al*.^28^. In the enzymatic synthesis of D-KDP and L-KDP, the reaction mixture (100 ml) consisted of 50 mM HEPES-NaOH buffer (pH 7.2), 10 mM D-xylonate (for D-KDP) or L-arabinonate (for L-KDP), and 1 mM MgCl_2_. After the addition of ~50 mg L-arabinonate dehydratase from *A. brasilense*^17^ (see Fig. S6e), the mixture was left at 30 °C overnight. D-KDP and L-KDP were purified by using the same procedure as L-KDR described previously^26^. An NMR analysis in D_2_O revealed that D- and L-KDP both existed as a rapidly interconverting mixture of the acyclic keto and cyclic furanose forms, as reported previously^30^.

### Enzyme assay

Acid-sugar dehydration activity was monitored by the semicarbazide method (end-point detection after 30 min at 30 °C) using 50 mM HEPES-NaOH buffer (pH 7.2) containing 10 mM of the substrate^51^; if necessary, 1 mM MgCl_2_ was added. This method was used for the assessment of substrate specificity and optimum pH for activity. As an alternative continuous method, D-arabinonate, D-xylonate, and L-arabinonate dehydratase activities were spectrophotometrically assayed at 340 nm in the coupling system with D-KDP 4-dehydrogenase (for the two former: C785_RS13675) or L-KDP 4-dehydrogenase (C785_RS20555). The reaction mixture consisted of 50 mM HEPES-NaOH buffer (pH 7.2) containing 1U of coupling enzymes and 1.5 mM NAD^+^. The reaction was started by the addition of 100 mM substrate (100 µl) with a final reaction volume of 1 ml. D- and L-KDP dehydratase activities were continuously assayed in 50 mM HEPES-NaOH buffer (pH 7.2) containing 10 mM substrate, and 1U αKGSA dehydrogenase^16^; if necessary, 1 mM MgCl_2_ was added. The reaction was started by the addition of 1.5 mM NAD^+^ solution (100 µl) with a final reaction volume of 1 ml. D- and L-KDP 4-dehydrogenase, aldose 1-dehydrogenase, and αKGSA dehydrogenase activities were assayed in 50 mM Tris-HCl (pH 9.0) containing 10 mM of the substrate. The reaction was initiated by the addition of 1.5 mM NAD(P)^+^ solution (100 µl) with a final reaction volume of 1 ml. One unit of all enzyme activities refers to 1 μmol NAD(P)H produced/min. *K*_m_ and *k*_cat_ values were calculated by a Lineweaver-Burk plot. 5-Hydroxy-2, 4-dioxo-pentanonate hydrolase activity was assessed in 50 mM HEPES-NaOH (pH 7.2) buffer containing 10 mM D-KDP, 1 mM MgCl_2_, and 10 mM NAD^+^. After the addition of 1 U purified C785_RS13675 (as D-KDP 4-dehydrogenase), the mixture was incubated at 30 °C for 10 min to form 5-hydroxy-2,4-dioxo-pentanonate. The reaction was started by the addition of a small amount of C785_RS20550 and analyzed by HPLC using an Aminex HPX-87H Organic Analysis column (Bio-Rad), as described previously^26^.

### Identification of reaction products

In order to remove glycerol in the storage buffer, the purified enzyme was dialyzed at 4 °C in several buffers: for C785_RS13685, 50 mM K_2_HPO_4_/KH_2_PO_4_ buffer (pH 7.0) containing 0.1 mM (NH_4_)_2_Fe(SO_4_)_2_·6H_2_O and 10 mM DTT; for C785_RS13680 and C785_RS13710, 50 mM K_2_HPO_4_/KH_2_PO_4_ buffer (pH 7.0) containing 1 mM MgCl_2_; for C785_RS13675, 50 mM K_2_HPO_4_/KH_2_PO_4_ buffer (pH 8.5). The next day, the enzyme solution was lyophilized, and solvated in D_2_O (1.2 ml) containing 20 mM D-arabinonate or D-altronate (for C785_RS13685), D-KDP (for C785_RS13680), L-KDP (for C785_RS13710), or D-KDP and NAD^+^ (for C785_RS13675). NMR spectra were recorded at 25 °C on a JEOL JNM-EC400 NMR spectrometer (JEOL Ltd., Tokyo, Japan) operating at 400 MHz. 2,2-Dimethyl-2-silapentane-5-sulfonate was used as an internal standard.

### Iron analysis of C785_RS13685 and C785_RS21250

The (potential) iron-sulfur cluster of C785_RS13685 and C785_RS21250 was analyzed by electron paramagnetic resonance (EPR) using a JEOL TE-300 X-band spectrometer operating with a 100-kHz field modulation. A temperature-dependent analysis was performed in the range of 10 to 40 K using a LTR-3 liquid helium cryostat (Air Products). The purified enzyme (~40 mg/ml) was dialyzed in 50 mM HEPES-NaOH (pH 7.2) containing 0.1 mM (NH_4_)_2_Fe(SO_4_)_2_·6H_2_O, 10 mM DTT, and 50% (v/v) glycerol (for C785_RS13685) or 50 mM HEPES-NaOH (pH7.2) containing 1 mM MgCl_2_ and 50% (v/v) glycerol (for C785_RS21250). If necessary, C785_RS21250 was anaerobically reduced with a 10-fold excess sodium dithionite (Na_2_S_2_O_4_). EPR spectra were recorded using the following representative conditions; microwave frequency 8.9820 to 8.9995 GHz monitored by internal frequency counter, microwave power; 5.0 mW, 100 kHz field modulation magnitude; 0.40 to 0.63 mT, center field; 280 ± 250 mT or 300 ± 50 mT, sweep time; 4.0 or 8.0 min, time constant; 0.1 sec, and receiver amplitude; 100 to 800. In the present study, *g*-values were evaluated based on the *g*-value of the Li-salt of tetracyanoquinodimethane (2.0025) as an external standard. The magnetic field strength of EPR spectra was calibrated using the hyperfine coupling constant (8.69 mT) of the Mn(II) ion doped in MgO powder.

### Growth and gene expression analyses

*H. huttiense* IAM 15032 (NBRC 102406) and *P. mimosarum* NBRC 106338 were cultured aerobically with vigorous shaking at 30 °C in nutrient medium containing 10 g peptone, 2 g yeast extract and MgSO_4_·7H_2_O per liter. Overnight-grown cells from 0.5 ml cultures were rinsed with a minimal medium, pH 6.8, containing 4.0 g KH_2_PO_4_, 6.0 g K_2_HPO_4_, 0.2 g MgSO_4_·7H_2_O, 0.1 g NaCl, 0.026 g CaSO_4_·2H_2_O, 1.0 g NH_4_Cl, 0.01 g FeCl_3_·6H_2_O, 0.002 g NaMoO_4_·2H_2_O, 0.0001 g biotin per liter, transferred to 3 ml aliqouts of the minimal medium supplemented with 1% (w/v) carbon sources, and then incubated at 30 °C for 4 h with vigorous shaking. The preparation of RNA samples and one-step real-time RT-PCR were performed as described previously^52^. The primers used for RT-PCR are listed in Table S2.

### Sequence comparison

Protein sequences were analyzed using the Protein-BLAST and Clustal W programs distributed by the DDBJ (DNA Data Bank of Japan).

## Electronic supplementary material

Supplementary Discussion

## References

[1] Peekhaus N, Conway T (1998). What’s for dinner?: Entner-Doudoroff metabolism in. Escherichia coli. J. Bacteriol..

[2] Bräsen C, Esser D, Rauch B, Siebers B (2014). Carbohydrate metabolism in Archaea: current insights into unusual enzymes and pathways and their regulation. Microbiol. Mol. Biol. Rev..

[3] Lamble HJ, Heyer NI, Bull SD, Hough DW, Danson MJ (2003). Metabolic pathway promiscuity in the archaeon *Sulfolobus solfataricus* revealed by studies on glucose dehydrogenase and 2-keto-3-deoxygluconate aldolase. J. Biol. Chem..

[4] Lamble HJ, Milburn CC, Taylor GL, Hough DW, Danson MJ (2004). Gluconate dehydratase from the promiscuous Entner-Doudoroff pathway in *Sulfolobus solfataricus*. FEBS Lett..

[5] Theodossis A (2004). The structural basis for substrate promiscuity in 2-keto-3-deoxygluconate aldolase from the Entner-Doudoroff pathway in *Sulfolobus solfataricus*. J. Biol. Chem..

[6] Shi NQ (2000). Characterization and complementation of a *Pichia stipitis* mutant unable to grow on D-xylose or L-arabinose. Appl. Biochem. Biotechnol..

[7] Entner N, Doudoroff M (1952). Glucose and gluconic acid oxidation of *Pseudomonas saccharophila*. J. Biol. Chem..

[8] Nunn CE (2010). Metabolism of pentose sugars in the hyperthermophilic archaea *Sulfolobus solfataricus* and *Sulfolobus acidocaldarius*. J. Biol. Chem..

[9] Wagner, M. *et al*. *Sulfolobus acidocaldarius* uptakes pentoses via a cut2-type ABC transporter and metabolizes them through the aldolase-independent Weimberg pathway. *Appl. Environ. Microbiol*. pii: AEM.01273-17 (2017). 10.1128/AEM.01273-17 PMC5772230 29150511

[10] Watanabe S, Saimura M, Makino K (2008). Eukaryotic and bacterial gene clusters related to an alternative pathway of non-phosphorylated L-rhamnose metabolism. J. Biol. Chem..

[11] Wolf J (2016). A systems biology approach reveals major metabolic changes in the thermoacidophilic archaeon *Sulfolobus solfataricus* in response to the carbon source L-fucose versus D-glucose. Mol. Microbiol..

[12] Stephens C (2007). Genetic analysis of a novel pathway for D-xylose metabolism in *Caulobacter crescentus*. J. Bacteriol..

[13] Johnsen U, Schönheit P (2004). Novel xylose dehydrogenase in the halophilic archaeon *Haloarcula marismortui*. J. Bacteriol..

[14] Johnsen U (2009). D-Xylose degradation pathway in the halophilic archaeon *Haloferax volcanii*. J. Biol. Chem..

[15] Watanabe S, Kodaki T, Makino K (2006). Cloning, expression and characterization of bacterial L-arabinose 1-dehydrogenase involved in an alternative pathway of L-arabinose metabolism. J. Biol. Chem..

[16] Watanabe S, Kodaki T, Makino K (2006). A novel α-ketoglutaric semialdehyde dehydrogenase: evolutionary insight into an alternative pathway of bacterial L-arabinose metabolism. J. Biol. Chem..

[17] Watanabe S, Shimada N, Tajima K, Kodaki T, Makino K (2006). Identification and characterization of L-arabonate dehydratase, L-2-keto-3-deoxyarabonate dehydratase and L-arabinolactonase involved in an alternative pathway of L-arabinose metabolism: novel evolutionary insight into sugar metabolism. J. Biol. Chem..

[18] Johnsen U, Sutter JM, Zaiß H, Schönheit P (2013). L-Arabinose degradation pathway in the haloarchaeon *Haloferax volcanii* involves a novel type of L-arabinose dehydrogenase. Extremophiles.

[19] Brouns SJ (2006). Identification of the missing links in prokaryotic pentose oxidation pathways: evidence for enzyme recruitment. J. Biol. Chem..

[20] Brouns SJ, Turnbull AP, Willemen HL, Akerboom J, van der Oost J (2007). Crystal structure and biochemical properties of the D-arabinose dehydrogenase from *Sulfolobus solfataricus*. J. Mol. Biol..

[21] Brouns SJ (2008). Structural insight into substrate binding and catalysis of a novel 2-keto-3-deoxy-D-arabinonate dehydratase illustrates common mechanistic features of the FAH superfamily. J. Mol. Biol..

[22] Vanfossen AL, Verhaart MR, Kengen SM, Kelly RM (2009). Carbohydrate utilization patterns for the extremely thermophilic bacterium *Caldicellulosiruptor saccharolyticus* reveal broad growth substrate preferences. Appl. Environ. Microbiol..

[23] Watanabe S, Yamada M, Ohtsu I, Makino K (2007). α-Ketoglutaric semialdehyde dehydrogenase isozymes involved in metabolic pathways of D-glucarate, D-galactarate and hydroxy-L-proline: molecular and metabolic convergent evolution. J. Biol. Chem..

[24] Aghaie A (2008). New insights into the alternative D-glucarate degradation pathway. J. Biol. Chem..

[25] Taberman H (2014). Structure and function of a decarboxylating *Agrobacterium tumefaciens* keto-deoxy-D-galactarate dehydratase. Biochemistry.

[26] Watanabe S, Makino K (2009). Novel modified version of non-phosphorylated sugar metabolism: an alternative L-rhamnose pathway of *Sphingomonas* sp. FEBS J..

[27] Bae J, Kim SM, Lee SB (2015). Identification and characterization of 2-keto-3-deoxy-L-rhamnonate dehydrogenase belonging to the MDR superfamily from the thermoacidophilic bacterium *Sulfobacillus thermosulfidooxidans*: implications to L-rhamnose metabolism in archaea. Extremophiles.

[28] Yew WS (2006). Evolution of enzymatic activities in the enolase superfamily: L-fuconate dehydratase from *Xanthomonas campestris*. Biochemistry.

[29] Felux AK, Spiteller D, Klebensberger J, Schleheck D (2015). Entner-Doudoroff pathway for sulfoquinovose degradation in *Pseudomonas putida* SQ1. Proc. Natl. Acad. Sci. USA.

[30] Archer RM (2013). Syntheses of 2-keto-3-deoxy-D-xylonate and 2-keto-3-deoxy-L-arabinonate as stereochemical probes for demonstrating the metabolic promiscuity of *Sulfolobus solfataricus* towards D-xylose and L-arabinose. Chemistry.

[31] Wichelecki DJ (2014). Enzymatic and structural characterization of rTSγ provides insights into the function of rTSβ. Biochemistry.

[32] Dreyer JL (1987). The role of iron in the activation of mannonic and altronic acid hydratases, two Fe-requiring hydro-lyases. Eur. J. Biochem..

[33] Andberg M (2016). Characterization and mutagenesis of two novel iron-sulphur cluster pentonate dehydratases. Appl. Microbiol. Biotechnol..

[34] Rahman MM (2017). The crystal structure of a bacterial L-arabinonate dehydratase contains a [2Fe-2S] cluster. ACS Chem. Biol..

[35] Rahman MM, Andberg M, Koivula A, Rouvinen J, Hakulinen N (2018). The crystal structure of D-xylonate dehydratase reveals functional features of enzymes from the Ilv/ED dehydratase family. Sci. Rep..

[36] Smiley DS, Ashwell G (1960). Uronic acid metabolism in bacteria. III. Purification and properties of D-altronic acid and D-mannonic acid dehydrases in *Escherichia coli*. J. Biol. Chem..

[37] Shimizu T, Takaya N, Nakamura A (2012). An L-glucose catabolic pathway in *Paracoccus* species 43P. J. Biol. Chem..

[38] Rein U (2005). Dissimilation of cysteate via 3-sulfolactate sulfo-lyase and a sulfate exporter in *Paracoccus pantotrophus* NKNCYSA. Microbiology.

[39] Groninger-Poe FP (2014). Evolution of enzymatic activities in the enolase superfamily: galactarate dehydratase III from *Agrobacterium tumefaciens* C58. Biochemistry.

[40] Pircher H (2015). Identification of FAH domain-containing protein 1 (FAHD1) as oxaloacetate decarboxylase. J. Biol. Chem..

[41] Ran T (2013). Crystal structures of Cg1458 reveal a catalytic lid domain and a common catalytic mechanism for the FAH family. Biochem. J..

[42] Ghasempur S (2014). Discovery of a novel L-lyxonate degradation pathway in *Pseudomonas aeruginosa* PAO1. Biochemistry.

[43] van de Werken HJ (2008). Hydrogenomics of the extremely thermophilic bacterium *Caldicellulosiruptor saccharolyticus*. Appl. Environ. Microbiol..

[44] Bartkus JM, Mortlock RP (1986). Isolation of a mutation resulting in constitutive synthesis of L-fucose catabolic enzymes. J. Bacteriol..

[45] Elsinghorst EA, Mortlock RP (1994). Molecular cloning of the *Escherichia coli* B L-fucose-D-arabinose gene cluster. J. Bacteriol..

[46] Yamamoto-Otake H, Nakano E, Koyama Y (1994). Cloning and sequencing of the L-fucose dehydrogenase gene from *Pseudomonas* sp. No. 1143. Biosci. Biotechnol. Biochem..

[47] Hobbs ME (2013). Discovery of an L-fucono-1,5-lactonase from cog3618 of the amidohydrolase superfamily. Biochemistry.

[48] Kobayashi Y, Ohtsu I, Fujimura M, Fukumori F (2011). A mutation in dnaK causes stabilization of the heat shock sigma factor σ32, accumulation of heat shock proteins and increase in toluene-resistance in *Pseudomonas putida*. Environ. Microbiol..

[49] Hishinuma S, Yuki M, Fujimura M, Fukumori F (2006). OxyR regulated the expression of two major catalases, KatA and KatB, along with peroxiredoxin, AhpC in *Pseudomonas putida*. Environ. Microbiol..

[50] Moore S, Link KP (1940). Carbohydrate characterization. I. The oxidation of aldoses by hypoiodite in methanol. II. The identification of seven aldomonosaccharides as benzimidazole derivatives. J. Biol. Chem..

[51] MacGee J, Doudoroff M (1954). A new phosphorylated intermediate in glucose oxidation. J. Biol. Chem..

[52] Ito F, Tamiya. T, Ohtsu I, Fujimura M, Fukumori F (2014). Genetic and phenotypic characterization of the heat shock response in *Pseudomonas putida*. Microbiology Open.

