# Supplementary material for: Novel non-phosphorylative pathway of pentose metabolism from bacteria
Source: Sci Rep. 2019 Jan 17;9:155. doi: 10.1038/s41598-018-36774-6 (PMC6336799; doi:10.1038/s41598-018-36774-6)
Supplement: Supplementary file 1 — Supplementary Discussion [file 41598_2018_36774_MOESM1_ESM.pdf]

Supplementary information

Novel non-phosphorylative pathway of pentose metabolism from bacteria

Seiya Watanabe[1,2,3], Fumiyasu Fukumori[4], Hisashi Nishiwaki[1,2], Yasuhiro Sakurai[5], Kunihiko Tajima[5], Yasuo Watanabe[1,2]

[1] Department of Bioscience, Graduate School of Agriculture, Ehime University, 3-5-7 Tarumi, Matsuyama, Ehime 790-8566, Japan
[2] Faculty of Agriculture, Ehime University, 3-5-7 Tarumi, Matsuyama, Ehime 790-8566, Japan
[3] Center for Marine Environmental Studies (CMES), Ehime University, 2-5 Bunkyo-cho, Matsuyama, Ehime 790-8577, Japan
[4] Faculty of Food and Nutritional Sciences, Toyo University, 1-1-1 Izumino, Itakura-machi, Ora-gun, Gunma 374-0193, Japan
[5] Department of Bio-molecular Engineering, Graduate School of Science and Technology, Kyoto Institute of Technology, Matsugasaki, Sakyo-ku, Kyoto 606-8585, Japan

Correspondence and requests for materials should be addressed to S.W. (e-mail: irab@agr.ehime-u.ac.jp).

Supplementary Discussion

Putative gene clusters related to pentose metabolism from *H. huttiense* IAM 15032

H. huttiense IAM 15032 possesses at least six interesting gene clusters.

Cluster 5. The combination of the protein superfamilies of the metabolic genes (enzymes) is the same as the typical ED pathway (Fig. 1c). C785_RS20885 is clearly related to the 6-phosphogluconate dehydratase involved in this pathway (Fig. S7e).

Cluster 6. The sulfoquinovose (6-deoxy-6-sulfoglucose) pathway from *Pseudomonas putida* SQ1 (1) is analogous to the non-phosphorylative ED pathway, and contains four enzymes catalyzing the schematic reactions A (PpSQ1_00089), B (PpSQ1_00091), C (PpSQ1_00089), and H (PpSQ1_00100). Furthermore, 3-sulfolactaldehyde, one of the metabolic products, is subsequently converted to 3-sulfolactate by 3-sulfolactaldehyde dehydrogenase (PpSQ1_00088). These genes are clustered on this bacterial genome. C785_RS04115, C785_RS04075, C785_RS04100, and C785_RS04080 show sequential identities of 51%, 51%, 62%, and 62% with PpSQ1_00089, PpSQ1_00091, PpSQ1_00089, and PpSQ1_00088, respectively.

Clusters 3 and 4. C785_RS21220 is sequentially similar to L-fuconate dehydratase from *X. campestris*; identity of 57% (2). C785_RS21250 and C785_RS00855 are sequentially similar to ILDV/EDD-like pentonate dehydratases (Fig. S7e) (3, 4) (see below). C785_RS00860 and C785_RS21245 are sequentially similar to D-xylose 1-dehydrogenase from *C. crescentus* (CC_0821); identities of 62% and 53%, respectively (Fig. S5).

Cluster 1. COG3970 (C785_RS13680 and C785_RS13710) and COG1028 (C785_RS13675) proteins (genes) are clustered with COG2721 (C785_RS13685), which is not contained in Figure 1c. Only the C-terminal halves of C785_RS13680 and C785_RS13710 show weak similarities to D-KDP dehydratase from Archaeon *S. solfataricus* (30%> identity) (5, 6). C785_RS13675 is sequentially similar to L-KDF 4-dehydrogenase from *X. campestris* (XCC4067); identity of 58% (2).

Cluster 2. C785_RS20550 and C785_RS20555 are sequentially similar to L-KDR 4-dehydrogenase (SKA58_03590) and 2,4-dioxo-pentanonate hydrolase (SKA58_03585) from *Sphingomonas* sp.; identities of 51% and 56%, respectively (7).

Putative lactone-sugar hydrolase genes. Based on Figure 1c, C785_RS13670, C785_RS21190, C785_RS21230, and C785_RS00850 in clusters 1, 3, and 4 appear to encode the lactone-sugar hydrolase catalyzing the schematic reaction B. Among them, C785_RS21190 and C785_RS21230 are clearly similar to L-fucono-1,5-lactonase from *X. campestris* and L-arabinolactonase from *A. brasilense*; identities of 37% and 53%, respectively (3, 8). However, we did not focus on these hypothetical genes in the present study because the lactone-sugar is non-enzymatically converted to the corresponding acid-sugar.

Based on these analyses, we speculated that clusters 1~4 are related to the non-phosphorylative pentose pathway(s).

Phylogenetic analysis of C785_RS21250 and C785_RS00855

The ILVD/EDD superfamily (COG0129) consists of three dehydratase enzymes that utilize different substrates: 6-phosphogluconate dehydratase (EC 4.2.1.12), dihydroxyacid dehydratase (EC 4.2.1.9), and pentonate dehydratase. The latter may be further classified into two subgroups. In the first, (a subgroup of) D-xylonate dehydratase (EC 4.2.1.82) from *C. crescentus* (CC_0819) exhibits significant activity not only for D-xylonate (a natural substrate), but also D-gluconate, but no activity for L-arabinonate (4). In the second, (a

subgroup of) L-arabinonate dehydratase (EC 4.2.1.25) from *A. brasilense* (BAE94269) strictly utilizes L-arabinonate as a substrate (3). C785_RS00855 and C785_RS21250 belong to the first and second subgroups, respectively, conforming with their substrate specificities (Fig. 2a and Fig. S7).

Phylogenetic analysis of the SDR protein superfamily (COG1028).

D-Xylose 1-dehydrogenase from *C. crescentus* (CC_0821) is a strict preference for D-xylose (9). Although L-arabinose is also active as a substrate, the V_{max}/K_m value is ~294-fold lower than that for D-xylose. As described above, since C785_RS00860 shows high sequence identity with CC_0821 (62%), the preference for D-xylose is reasonable; the ratio of D-xylose to L-arabinose in k_{cat}/K_m values is ~52 (Table S2). Although C785_RS21245 also shows significant sequence similarity to C785_RS00855 (and CC_0821) (48%), substrate specificity is clearly preferential for L-arabinose; the ratio of L-arabinose to D-xylose in k_{cat}/K_m values is ~14 (Table S2). We previously revealed that L-arabinose 1-dehydrogenase from *A. brasilense* (10) is one of the members of the glucose-fructose oxidoreductase superfamily (pfam01408). On the other hand, the enzyme from Archaeon *Haloferax volcanii* DS2 (HVO_B0032) belongs to the same SDR superfamily as C785_RS21245 (11), whereas there is a poor phylogenetic relationship between them (Fig. S5), indicating their convergent evolution. Therefore, to the best of our knowledge, this is the first study to show a novel type of L-arabinose 1-dehydrogenase from bacteria.

Although C785_RS21215 shows a poor phylogenetic relationship to any subgroup of the SDR superfamily (Fig. S5), 25 acid sequence residues at the N terminus are significantly similar to that of L-fucose 1-dehydrogenase from *Acinetobacter* sp. strain SA-134; MDYHEKNKVFIVTGGGAGIGGAIS (underlined letters indicate the same amino acid residues as C785_RS21215) (12). Although a gene encoding this enzyme has not been cloned, a hypothetical BSR55_RS03540 protein from *Acinetobacter bereziniae* strain XH901 possesses a significantly similar N-terminal amino acid sequence to L-fucose 1-dehydrogenase from *Acinetobacter* sp. strain SA-134 (MDLHLKNKVFIVTGGGAGIGGAIS, underlined letters). There is a sequence identity of 60% between C785_RS21215 and BSR55_RS03540. On the other hand, L-fucose 1-dehydrogenase from *Pseudomonas* sp. No. 1143 belongs to the aldo/keto reductase superfamily (COG0667), and D-arabinose is active as a substrate (13). Although *H. huttiense* IAM 15032 possesses the homologous gene, C785_RS09855, L-fucose exerted no effect on transcription, and the recombinant protein showed no activity for L-fucose (data not shown). Based on these results, we speculated that C785_RS21215 (but not C785_RS09855) encodes a novel type of L-fucose 1-dehydrogenase; however, the k_{cat}/K_m value for L-fucose (0.0126 $min^{-1}\cdot mM^{-1}$) using the recombinant protein was extremely low (Table S1 and Fig. 5b).

Complementation of D-arabinose-defective *E. coli*

To express multiple metabolic genes involved in non-phosphorylative D-arabinose metabolism (Route II or III) in *E. coli* cells, three plasmid vectors, pCOLADuet, pETDuet, and pACYCDuet (Novagen; each vector carries compatible replicons and antibiotic resistant makers), and *E. coli* BL21(DE3) were used: BAF33385 gene from *Azospirillum brasilense* NBRC 102289 was used as αKGSA dehydrogenase gene (14). The overall scheme of plasmid construction and the primers used are shown in Fig. S8a and Table S2, respectively. Briefly, the C785_RS13685 (encoding to D-arabinonate dehydratase), C785_RS13680 (D-KDP

dehydratase), C785_RS13675 (D-KDP dehydrogenase), C785_RS20550 genes (5-hydroxy-2,4-dioxo-pentanonate hydrolase) were amplified using each set of specific primers and introduced into the BamHI-HindIII sites in the multiple cloning site (MCS) 1 of pACYCDuet-1 (for C785_RS13685 gene), pETDuet-1 (for C785_RS13680 and C785_RS13675 genes), or pCOLADuet-1 (for C785_RS20550 and BAF33385 genes) (encoding $(His)_6$-tag sequences at the N-terminus of the expressed protein) to obtain plasmids pACYC/C785_RS13685, pET/C785_RS13680, pET/C785_RS13675, pCOLA/C785_RS20550, and pCOLA/BAF33385, respectively. On the other hand, the A19U_RS0129385 gene (D-arabinose 1-dehydrogenase) was amplified as DNA fragment containing N-terminal $(His)_6$-tag sequences using the pQE-80L plasmid containing this gene, and introduced into the NdeI-XhoI sites in MCS 2 of pACYC/C785_RS13685 to obtain plasmids pACYC/C785_RS13685/A19U_RS0129385. These constructs allow all the introduced genes to be expressed as N-terminal $(His)_6$-tagged proteins. The purified plasmids constructs and/or empty Duet vectors were mixed appropriately and used to transform *E. coli* BL21(DE3) by a heat-shock method: among them, Ec13 and Ec16 possess a set of Routes II and III of the non-phosphorylative D-arabinose pathway, respectively.

All constructed recombinant *E. coli* strains could grow in M9 minimal medium containing 2% (w/v) D-glucose as a sole carbon source, but not D-arabinose. Western blot analysis using cell-free extract prepared from these *E. coli* strains revealed that most of the introduced metabolic genes may express functionally (Fig. S8b). To estimate their expression levels in more detail, all expressed $(His)_6$-tagged proteins were purified by using Ni-NTA spin column (QIAGEN) and analyzed by SDS-PAGE (Fig. S8c). As results, expression levels of C785_RS13685, C785_RS13680 and C785_RS13675 were comparable when each gene was expressed in *E. coli* DH5α, whereas that of A19U_RS0129385 (and C785_RS20550) was extremely low.

References

1. Felux, A. K., Spiteller, D., Klebensberger, J. & Schleheck, D. Entner-Doudoroff pathway for sulfoquinovose degradation in *Pseudomonas putida* SQ1. *Proc. Natl. Acad. Sci. U S A.* **112,** E4298-305 (2015).
2. Yew, W. S. *et al.* Evolution of enzymatic activities in the enolase superfamily: L-fuconate dehydratase from *Xanthomonas campestris*. *Biochemistry* **45,** 14582-14597 (2006).
3. Watanabe, S., Shimada, N., Tajima, K., Kodaki, T. & Makino, K. Identification and characterization of L-arabonate dehydratase, L-2-keto-3-deoxyarabonate dehydratase and L-arabinolactonase involved in an alternative pathway of L-arabinose metabolism: novel evolutionary insight into sugar metabolism. *J. Biol. Chem.* **281,** 33521-33536 (2006).
4. Andberg, M. *et al.* Characterization and mutagenesis of two novel iron-sulphur cluster pentonate dehydratases. *Appl. Microbiol. Biotechnol.* **100,** 7549-7563 (2016).
5. Brouns, S. J. *et al.* Identification of the missing links in prokaryotic pentose oxidation pathways: evidence for enzyme recruitment. *J. Biol. Chem.* **281,** 27378-27388 (2006).
6. Brouns, S. J. *et al.* Structural insight into substrate binding and catalysis of a novel 2-keto-3-deoxy-D-arabinonate dehydratase illustrates common mechanistic features of the FAH superfamily. *J. Mol. Biol.* **379,** 357-371 (2008).
7. Watanabe, S. & Makino, K. Novel modified version of non-phosphorylated sugar metabolism: an alternative L-rhamnose pathway of *Sphingomonas* sp. *FEBS J.* **276,** 1554-1567 (2009).

8. Hobbs, M. E. *et al.* Discovery of an L-fucono-1,5-lactonase from cog3618 of the amidohydrolase superfamily. *Biochemistry* **52,** 239-253 (2013).
9. Stephens, C. *et al.* Genetic analysis of a novel pathway for D-xylose metabolism in *Caulobacter crescentus*. *J. Bacteriol.* **189,** 2181-2185 (2007).
10. Watanabe, S., Kodaki, T. & Makino, K. Cloning, expression and characterization of bacterial L-arabinose 1-dehydrogenase involved in an alternative pathway of L-arabinose metabolism. *J. Biol. Chem.* **281,** 2612-2623 (2006).
11. Johnsen, U., Sutter, J. M., Zaiß, H. & Schönheit, P. L-Arabinose degradation pathway in the haloarchaeon *Haloferax volcanii* involves a novel type of L-arabinose dehydrogenase. *Extremophiles* **17,** 897-909 (2013).
12. Ohshiro, T. & Morita, N. Production and characterization of L-fucose dehydrogenase from newly isolated *Acinetobacter* sp. strain SA-134. *Prep. Biochem. Biotechnol.* **44,** 382-391 (2014).
13. Yamamoto-Otake, H., Nakano, E. & Koyama, Y. Cloning and sequencing of the L-fucose dehydrogenase gene from *Pseudomonas* sp. No. 1143. *Biosci. Biotechnol. Biochem.* **58,** 2281-2282 (1994).
14. Watanabe, S., Yamada, M., Ohtsu, I. & Makino, K. α-Ketoglutaric semialdehyde dehydrogenase isozymes involved in metabolic pathways of D-glucarate, D-galactarate and hydroxy-L-proline: molecular and metabolic convergent evolution. *J. Biol. Chem.* **282,** 6685-6695 (2007).

Table S1. Kinetic parameters for putative aldose 1-dehydrogenases.

Proteins	Substrates	Coenzyme	Specific activity (units/mg protein)	K_m (mM)	k_{cat} (min^{-1})	k_{cat}/K_m ($min^{-1} \cdot mM^{-1}$)
C785_RS00860	D-Xylose	NAD^+	58.6	0.585±0.162	2570±377	4530±726
	D-Glucose		39.1	1.38±0.19	827±89	602±18
	D-Fucose		43.0	5.70±1.36	1900±264	338±30
	L-Arabinose		20.6	27.5±3.5	2400±260	87.4±1.7
C785_RS21245	D-Fucose	NAD^+	59.5	0.0701±0.0109	1970±179	28300±2050
	L-Arabinose		143	0.449±0.017	4360±65	9720±255
	D-Xylose		89.9	5.75±0.23	4000±122	695±6
	D-Glucose		78.3	7.38±0.47	3860±223	498±2
	L-Fucose		27.9	10.7±2.7	1850±333	175±15
C785_RS21215	L-Fucose	$NADP^+$	0.316	6280±920	79.5±11.7	0.0126±0.0001
A19U_RS0129385	D-Arabinose	NAD^+	30.1	0.250±0.016	1180±27	4700±197
	L-Fucose		39.5	0.711±0.062	1510±85	2130±70

Table S2. Primers used in this study.

Primer	Sequence*
Cloning of the C785_RS13685 gene into pQE-80L	
P1 (BamHI)	5′-catggatccAAAGCCAACTCTCCCATCATCCGCC-3′
P2 (HindIII)	5′-attaagctTCAGGTGTAGACGCCGATGTGCCAG-3′
Cloning of the C785_RS13680 gene into pQE-80L	
P3 (BamHI)	5′-catggatccGCACACACTTTTTCGCTCCAGGCCC-3′
P4 (HindIII)	5′-attaagctTTAAACGAGTTTGCGATTGGCGAGATTC-3′
Cloning of the C785_RS13710 gene into pQE-80L	
P5 (BamHI)	5′-catggatccAGGCTGATTCAATATCGCGACCTGC-3′
P6 (HindIII)	5′-attaagcttTACAGCTGCCTGACGCCACCCGGTC-3′
Cloning of the A19U_RS0104695 gene into pQE-80L	
P7 (BamHI)	5′-catggatccACTACGAGCCGTACGCCCCGCTACC-3′
P8 (HindIII)	5′-attaagctTTCACTTACCCCAGCGCAGCACGAG-3′
Cloning of the C785_RS13675 gene into pQE-80L	
P9 (BamHI)	5′-catggaTCCGCATCCACTGGCCGCCTCGCCG-3′
P10 (HindIII)	5′-attaagctTTAGTTGGACCACCCACCATCAATC-3′
Cloning of the C785_RS20555 gene into pQE-80L	
P11 (BamHI)	5′-catggatccAATCACTACGATTTCCAGGGCCGC-3′
P12 (HindIII)	5′-attaagctTCAGTAGGTGGCTCGTCCGCCCGAG-3′
Cloning of the C785_RS20550 gene into pQE-80L	
P13 (BamHI)	5′-catggatccAAATTACTGCGTTACGGCCCGGTGG-3′
P14 (HindIII)	5′-attaagctTCAGGCGTCGATGGTCTTCTGCTGC-3′
Cloning of the C785_RS21250 gene into pQE-80L	
P15 (BamHI)	5′-catggaTCCAGCGACAAAAAAGACAAGAGCCGC-3′
P16 (HindIII)	5′-attaagctTCAGTGCGAGTGCTTGGGCACGGCC-3′
Cloning of the C785_RS00855 gene into pQE-80L	
P17 (BamHI)	5′-catggatccGCACACACTTTTTCGCTCCAGGCCC-3′
P18 (HindIII)	5′-attaagctTTAAACGAGTTTGCGATTGGCGAGATTC-3′
Cloning of the C785_RS21245 gene into pQE-80L	
P19 (BamHI)	5′-catggaTCCAACACTCCCCAGAACGTGCAACTGG-3′
P20 (HindIII)	5′-attaagctTCACACCCAGCCGGCGTCGACGATG-3′
Cloning of the C785_RS00860 gene into pQE-80L	
P21 (BamHI)	5′-catggatccACCGCAGCTACCCCCACCGTCTTCG-3′
P22 (HindIII)	5′-attaagctTTACGCGCCCAGCCAGCCGGCATCG-3′

Cloning of the C785_RS21215 gene into pQE-80L	
P23 (BamHI)	5′-catggatccGATTTGAACCTGCAAGACAAGGTCG-3′
P24 (HindIII)	5′-attaagctTCAGGTCAGGGCGCGGTCGAGATGG-3′
Cloning of the A19U_RS0129385 gene into pQE-80L	
P25 (BamHI)	5′-catggatccGAAATCCAGAAACGACTGTTCCGGC-3′
P26 (HindIII)	5′-attaagctTATCGCGGCGTGGGTTCGGGCGCG-3′
The C785_RS13710 gene in pUCP26KmAhpC$_p$	
P27 (XhoI)	5′-ccatctcgagCACCATCACCATCACCATGGATCC-3′
P28 (MfeI)	5′-gcttcaattgTCACCAATAAAAAACGCCCGGC-3′
Site-directed mutagenesis in the C785_RS13685 gene[§]	
P29 (C134S)	5′-CCTCGGTGAACaGCTCGGCCACCG-3′
P30 (C173S)	5′-CACGGCCAGGGTaGCGCCACGGATTC-3′
P31 (C208S)	5′-GTGGGCCTGGGCaGCGAGACCAACC-3′
P32 (C276S)	5′-CTGGGCCTGCAGaGCGGTGGTTCGG-3′
P33 (C344S)	5′-GGGAGGAGTATaGCGCCAAGAACG-3′
P34 (C423S)	5′-GCCAACATGATCaGCTTCACCACGG-3′
P35 (C434S)	5′-CGGCCTATGGCaGCGCACCGGCAC-3′
P36 (C460S)	5′-CATGGACATCAACaGCGGCGAGATTG-3′
qRT-PCR of the C785_RS13675 gene	
P37	5′-TATTGGTCGCGCCTCGACTG-3′
P38	5′-CGAGCTCCTCCAGATGCGT-3′
qRT-PCR of the C785_RS13680 gene	
P39	5′-ACGCTGATCGGCCGCCTCT-3′
P40	5′-AGCGTGAGGTCATACGCACCGTC-3′
qRT-PCR of the C785_RS13685 gene	
P41	5′-TGAAAGCCAACTCTCCCATCATCC-3′
P42	5′-GCACGGTGCCGGAGATGAG-3′
qRT-PCR of the C785_RS13710 gene	
P43	5′-GGTCGATACCATGCGCGAA-3′
P44	5′-AGCGGTCGGTATTGAGCAG-3′
qRT-PCR of the C785_RS20550 gene	
P45	5′-CGGCGTCCTCGACCAATCC-3′
P46	5′-CCAGGGAAGCGTCATCCAGT-3′
qRT-PCR of the C785_RS20555 gene	
P47	5′-CAAGGCCAAAGTGGTGCTGT-3′

P48	5′-CGACATTGCCCAGCGGTTC-3′
qRT-PCR of the C785_RS21190 gene	
P49	5′-CCATCAGCATTTCTGGCGCTATC-3′
P50	5′-GACGATCACAGGCCAGCAGT-3′
qRT-PCR of the C785_RS21215 gene	
P51	5′-TGCAAGACAAGGTCGTCATCGT-3′
P52	5′-ACCACGGGAATTGCGCCTT-3′
qRT-PCR of the C785_RS21220 gene	
P53	5′-GTACGCTTCCCCACTTCGCAA-3′
P54	5′-TGTCGGTATCGAGGATGACGTAGG-3′
qRT-PCR of the C785_RS21245 gene	
P55	5′-GGCGAACTTCCCCAGCCTC-3′
P56	5′-CCTTGTTGCGCGAACGCTT-3′
qRT-PCR of the C785_RS21250 gene	
P57	5′-GTACCGCCGACAAGAACGGTTT-3′
P58	5′-ATGACCGGCTTGCCCTGGA-3′
qRT-PCR of the C785_RS00850 gene	
P59	5′-CCGGTGAGAAGGATCAAGGGG-3′
P60	5′-CGATCCACGGATAATCCTCGGAA-3′
qRT-PCR of the C785_RS00855 gene	
P61	5′-CGACATGACGGCGCTCTAC-3′
P62	5′-GGAGATGCCGATGATGGGAC-3′
qRT-PCR of the C785_RS00860 gene	
P63	5′-CGAACACGCCCTCTATCGCAG-3′
P64	5′-CCCGACGAAGGCTTCCACC-3′
qRT-PCR of the A19U_RS0129385 gene	
P65	5′-TCTCGGCAACCTCTTTTCAGTC-3′
P66	5′-CGAAATATCTGACTCCGCTGTCC-3′
qRT-PCR of the A19U_RS0129390 gene	
P67	5′-CAGCTTGTGGGTGGCACAAC-3′
P68	5′-CGTGAGGCGACCTTGTGACC-3′
qRT-PCR of the A19U_RS0129395 gene	
P69	5′-GCTGCCGTTTGATCTGGACT-3′
P70	5′-GCGATCACACATGGGCCTT-3′
qRT-PCR of the A19U_RS0100660 gene	

P71	5′-GCACGGCGCTGGAAGTGAG-3′
P72	5′-CTCGGAGAGGTCGCGATACAAAC-3′
qRT-PCR of the A19U_RS0100680 gene	
P73	5′-GACGCTCAGGGCCGCATTC-3′
P74	5′-CCGCGTCCGAGAGCACATC-3′
qRT-PCR of the A19U_RS0104685 gene	
P75	5′-GCTTCATGTACCGAAGCTGG-3′
P76	5′-CGTGTTGCAGATGCCGATGA-3′
qRT-PCR of the A19U_RS0104695 gene	
P77	5′-CAAGCCAGAAACGCGCTGT-3′
P78	5′-AGAGCGAGAACTGCTCCGAA-3′
qRT-PCR of the A19U_RS0115290 gene	
P79	5′-AAACACGGGCCCCTCTGTC-3′
P80	5′-TCGAAAAGGTCTGCGGTGGT-3′
qRT-PCR of the A19U_RS0115295 gene	
P81	5′-GTCGAGCGCTTCATGAACTATGG-3′
P82	5′-CGCAATGCCGATGATGGGAC-3′
Cloning of the C785_RS13685 gene into pACYCDuet-1	
P83 (BamHI)	5′-cat<u>ggatcc</u>gAAAGCCAACTCTCCCATCATCCGCC-3′
P84 (HindIII)	The same as P2.
Cloning of the C785_RS13680 gene into pETDuet-1	
P85 (BamHI)	5′-cat<u>ggatcc</u>gGCACACACTTTTTCGCTCCAGGCCC-3′
P86 (HindIII)	The same as P4.
Cloning of the C785_RS13675 gene into pETDuet-1	
P87 (BamHI)	5′-cat<u>ggatcc</u>gTCCGCATCCACTGGCCGCCTCGCCG-3′
P88 (HindIII)	The same as P10.
Cloning of the C785_RS20550 gene into pCOLADuet-1	
P89 (BamHI)	5′-cat<u>ggatcc</u>gAAATTACTGCGTTACGGCCCGGTGG-3′
P90 (HindIII)	The same as P14.
Cloning of the BAF33385 gene into pCOLADuet-1	
P91 (BamHI)	5′-cat<u>ggatcc</u>gCAACTGACTGGAGAGATGCTGATCG-3′
P92 (HindIII)	5′-ctaatt<u>aagctT</u>CACTCGGCCTTCCCGTCCCGCAGCCGCG-3′
Cloning of the A19U_RS0129385 gene into pACYC/C785_RS13685	
P93 (NdeI)	5′-gtcgac<u>catatg</u>ATGAGAGGATCTCACCATCACCATCACC-3′
P94 (XhoI)	5′-att<u>ctcgag</u>CTATCGCGGCGTGGGTTCGGGCGCG-3′

*Lower case letters indicate additional bases for introducing the digestion sites of restriction enzymes in parentheses.

§Only sense primers are shown. Underlining indicates mutated regions.

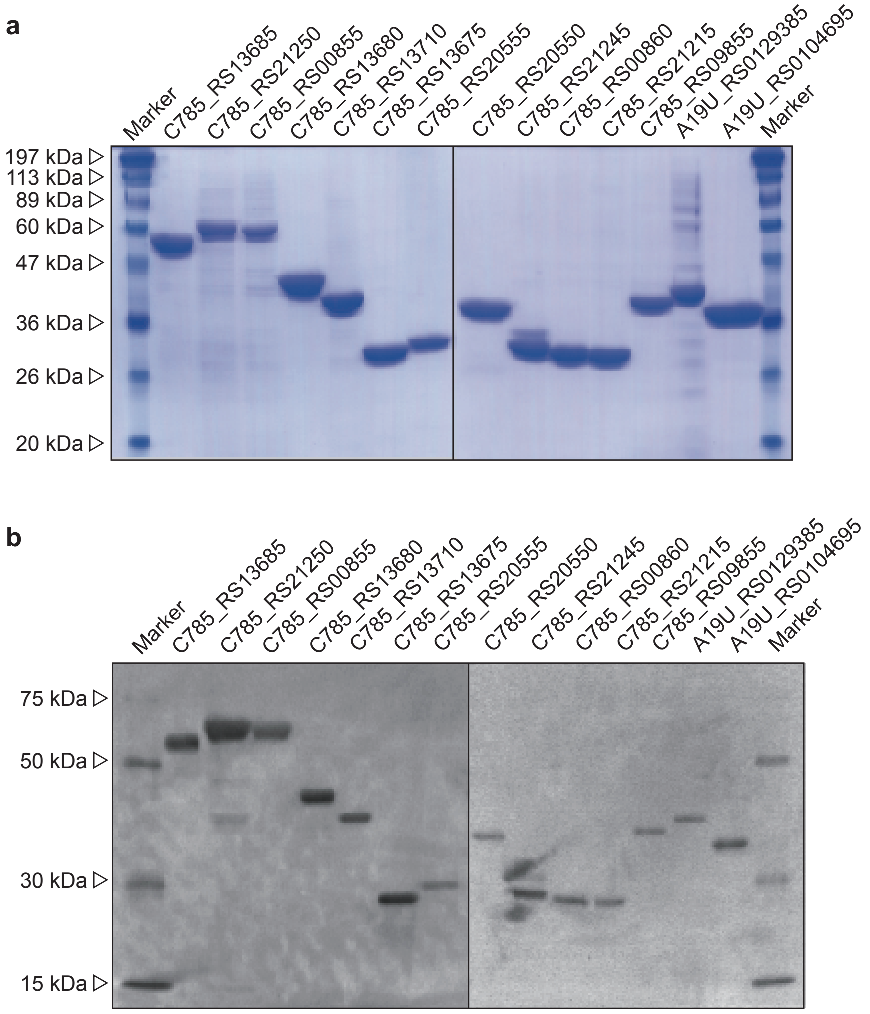

Figure S1. SDS-PAGE (a) and western blot analysis (b) of purified recombinant proteins. (**a**) Five micrograms each of the purified protein were applied to a 12% (w/v) gel. (**b**) After SDS-PAGE of 5 µg protein per lane, antibodies against an anti-(His)$_6$-tag antibody were used for immunoblotting. The PerfectPro 6×His-tagged Protein Ladder (Funakoshi) was used as a marker.

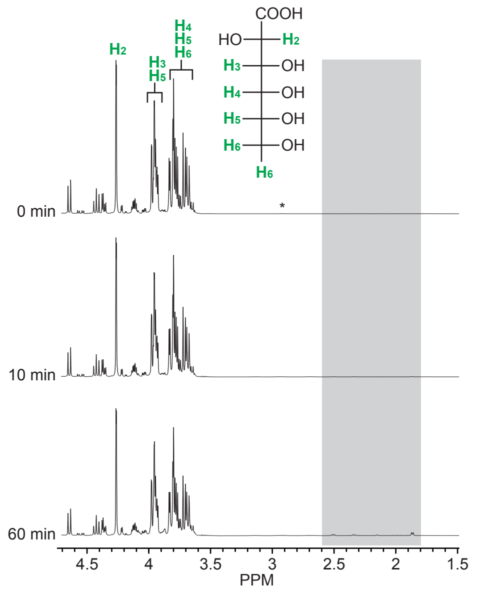

Figure S2. ^{1}H NMR spectra of the dehydration of D-altronate by C785_RS13685 in D_2O. The gray region was expanded in Figure 2e. Asterisks are peaks derived from an internal standard.

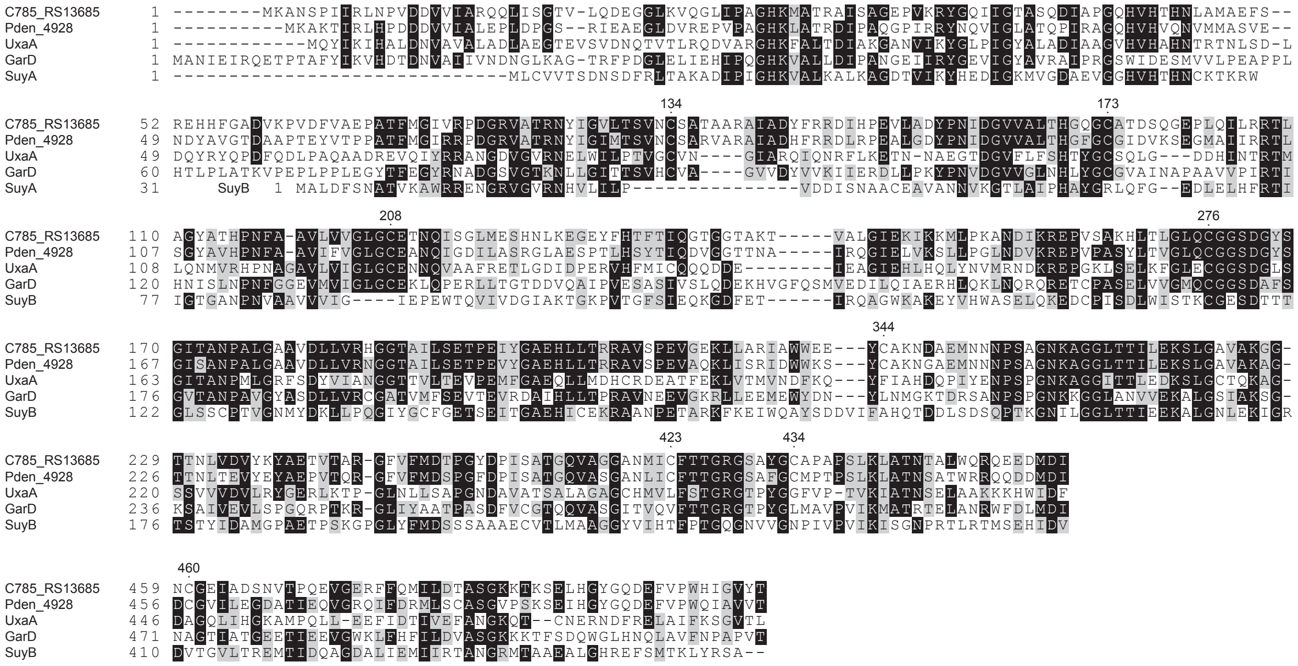

Figure S3. Multiple amino acid sequence alignments of UxaA/GarD superfamily members. Cysteine residues with numbers in C785_RS13685 were substituted to serine. White letters in black boxes indicate highly conserved amino acids.

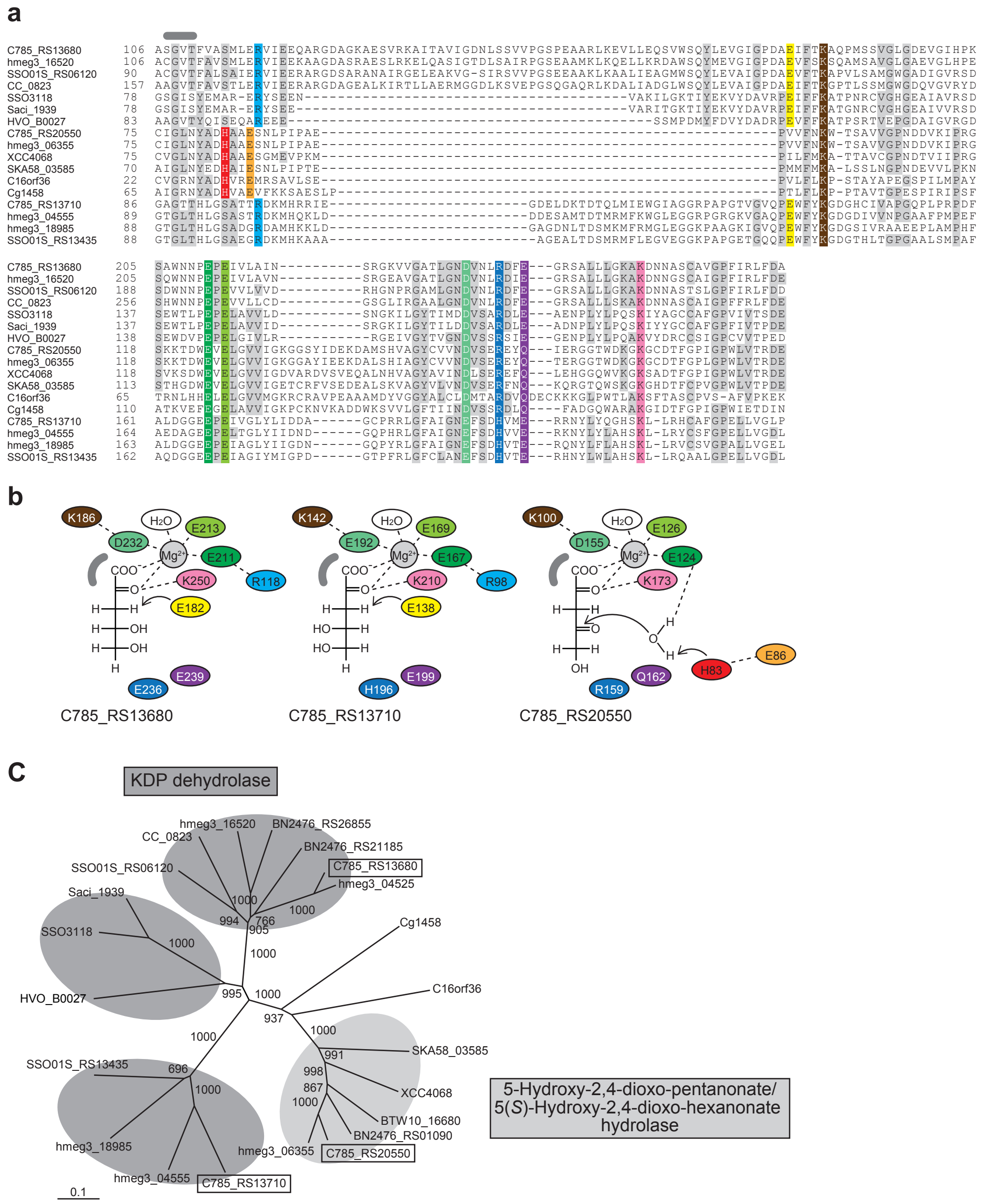

Figure S4. Phylogenetic analysis of the FAH/MhpD superfamily. (**a**) Partial multiple amino acid sequence alignments. Colors of active sites correspond to Figure S4b. (**b**) Schematic diagrams showing the predicted active sites of C785_RS13680, C785_RS13710, and C785_RS20550. (**c**) Phylogenetic relationship between C785_RS13680, C785_RS13710, C785_RS20550, and SSO3118. The number on each branch indicates the bootstrap value.

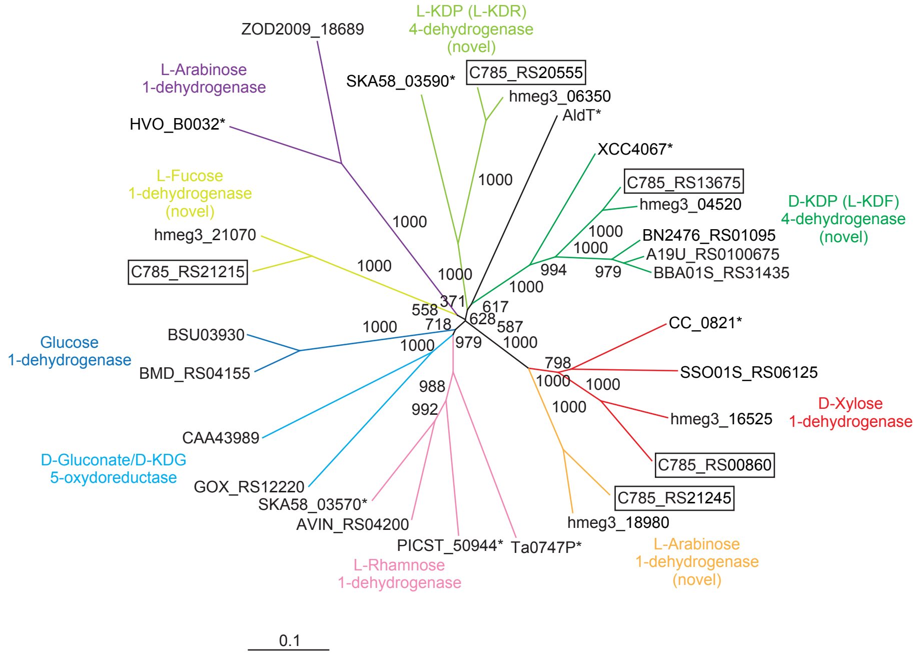

Figure S5. Phylogenetic tree of the SDR superfamily, including C785_RS21215, C785_RS21245, C785_RS00860, C785_RS13675, and C785_RS20555. Proteins with asterisks were functionally characterized. The number on each branch indicates the bootstrap value.

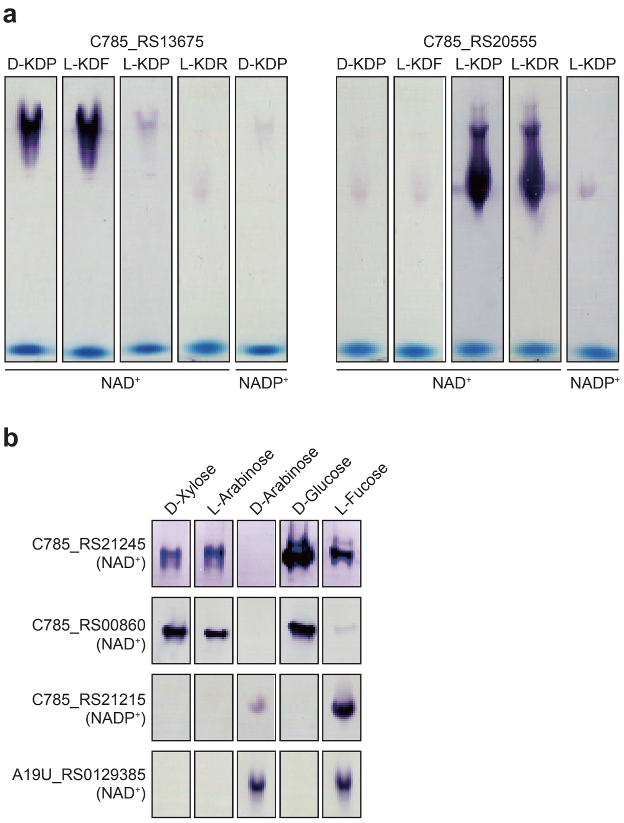

Figure S6. Zymogram staining analysis of KDP 4-dehydrogenases (a) and aldose 1-dehydrogenases (b). The purified enzyme was separated on non-denaturing PAGE with an 8% gel at 4°C. The gel was then soaked in staining solution consisting of 50 mM Tris-HCl (pH 9.0), 10 mM substrate, 0.25 mM nitroblue tetrazolium, 0.06 mM phenazine methosulfate, and 10 mM $NAD(P)^+$ at room temperature for 15 min. Dehydrogenase activity appeared as a violet band.

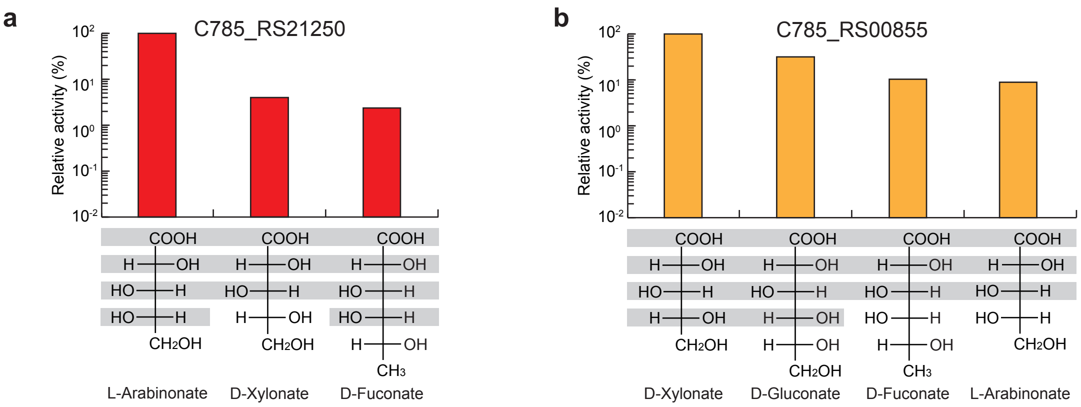

c

Substrates	Specific activity (unit/mg protein)	K_m (mM)	k_{cat} (min^{-1})	k_{cat}/K_m (min^{-1} mM^{-1})
L-Arabinonate	0.816±0.015	1.49±0.06	60.5±0.5	40.6±1.4

d

Substrates	Specific activity (unit/mg protein)	K_m (mM)	k_{cat} (min^{-1})	k_{cat}/K_m (min^{-1} mM^{-1})
D-Xylonate	5.68±0.12	0.192±0.003	401±3	2090±12
D-Gluconate	1.12±0.03	0.143±0.011	71.8±1.5	505±26

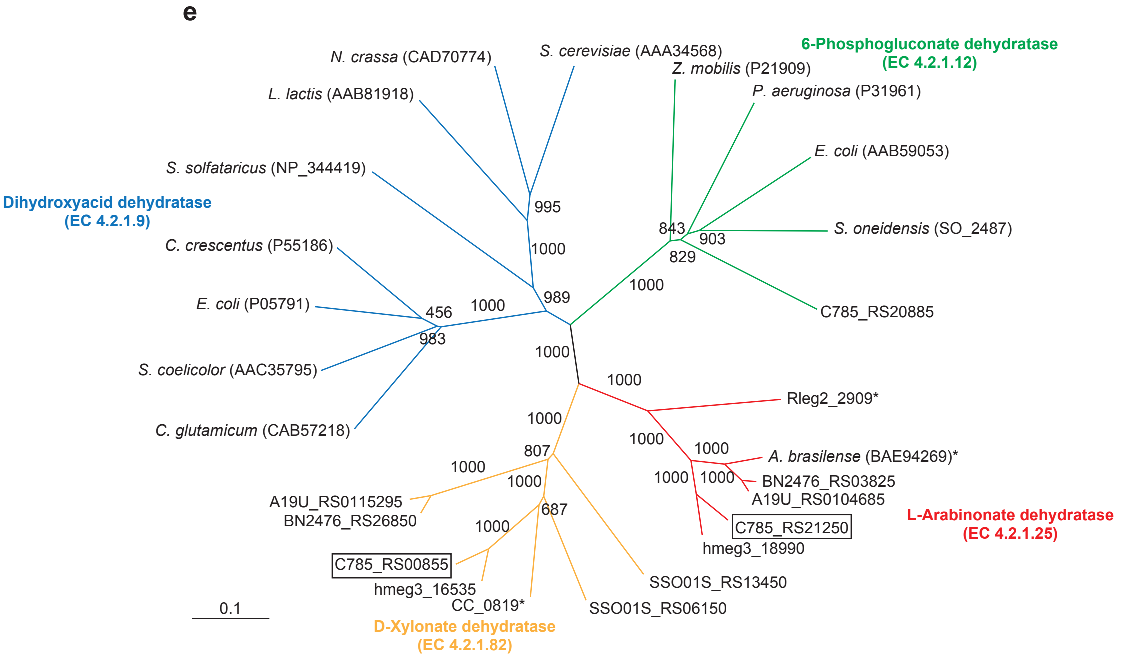

Figure S7. Functional characterization of C785_RS21250 and C785_RS00855. Substrate specificity (**a, b**) and kinetic parameters (**c, d**) of C785_RS21250 (**a, c**) and C785_RS00855 (**b, d**). Phylogenetic tree of the ILVD/EDD superfamily including C785_RS21250 and C785_RS00855. The number on each branch indicates the bootstrap value.

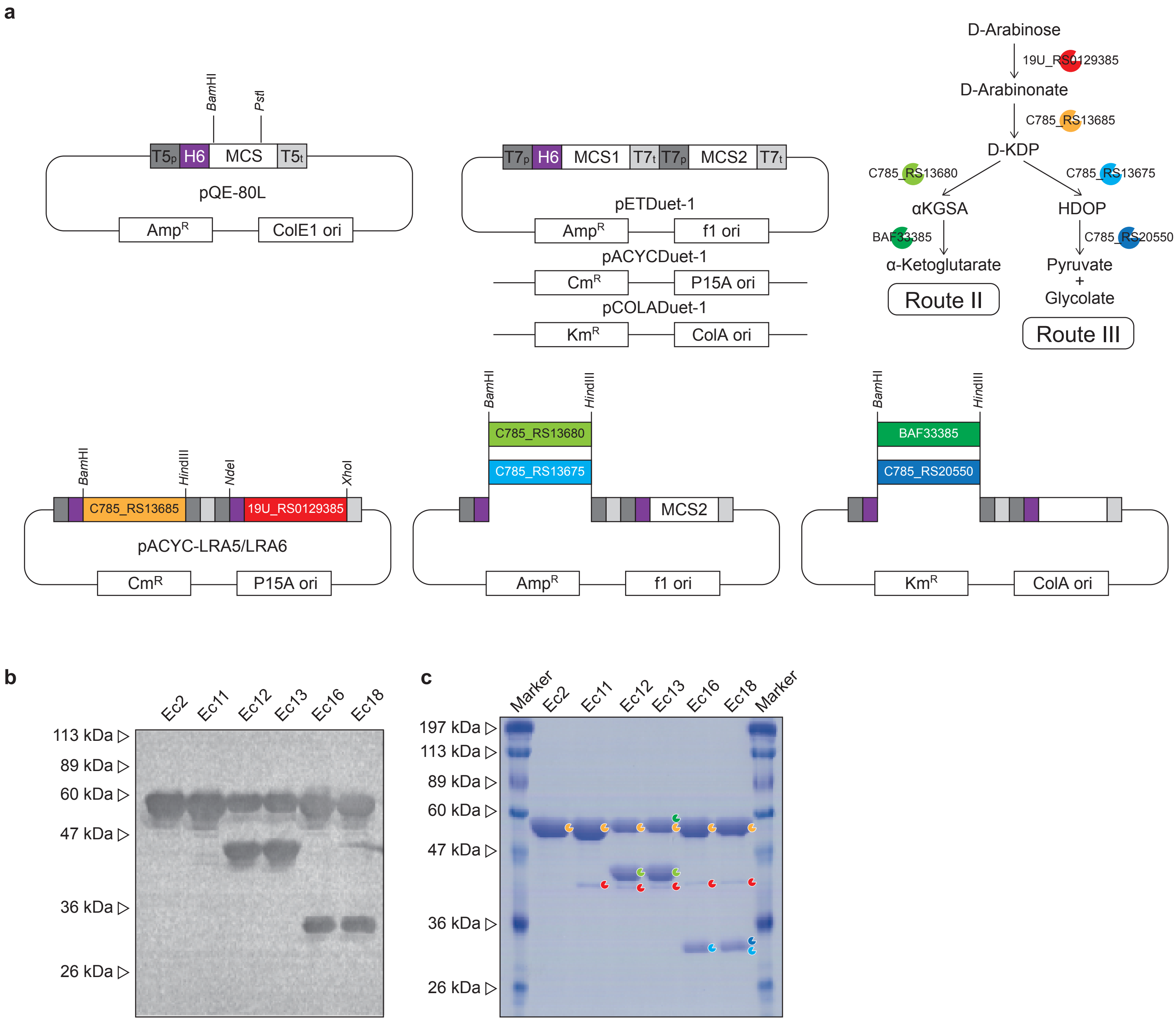

Supplementary Figure S8. Expression of metabolic genes related to D-arabinose pathway(s) in *E. coli*. (**a**) Overview of plasmid vectors used in this experiment. Three plasmid vectors, pETDuet-1, pACYCDuet-1, and pCOLADuet-1 have different replicons and antibiotics marker genes, respectively. Dark-gray and light-gray regions indicate the promoter and terminator, respectively. All genes were expressed as N-terminal $(His)_6$-tagged proteins (purple region). (**b**) Western blotting analysis. All enzymes were overexpressed as $(His)_6$-tagged proteins in *E. coli* cells grown in LB medium. Each of 100 µg of the cell-free extract was applied on 12% (w/v) gel. (**c**) SDS-PAGE. All $(His)_6$-tagged proteins in cell-free extract were purified by Ni^{2+}-chelating affinity column. Each of 50 µg of the purified proteins was applied on 12% (w/v) gel.

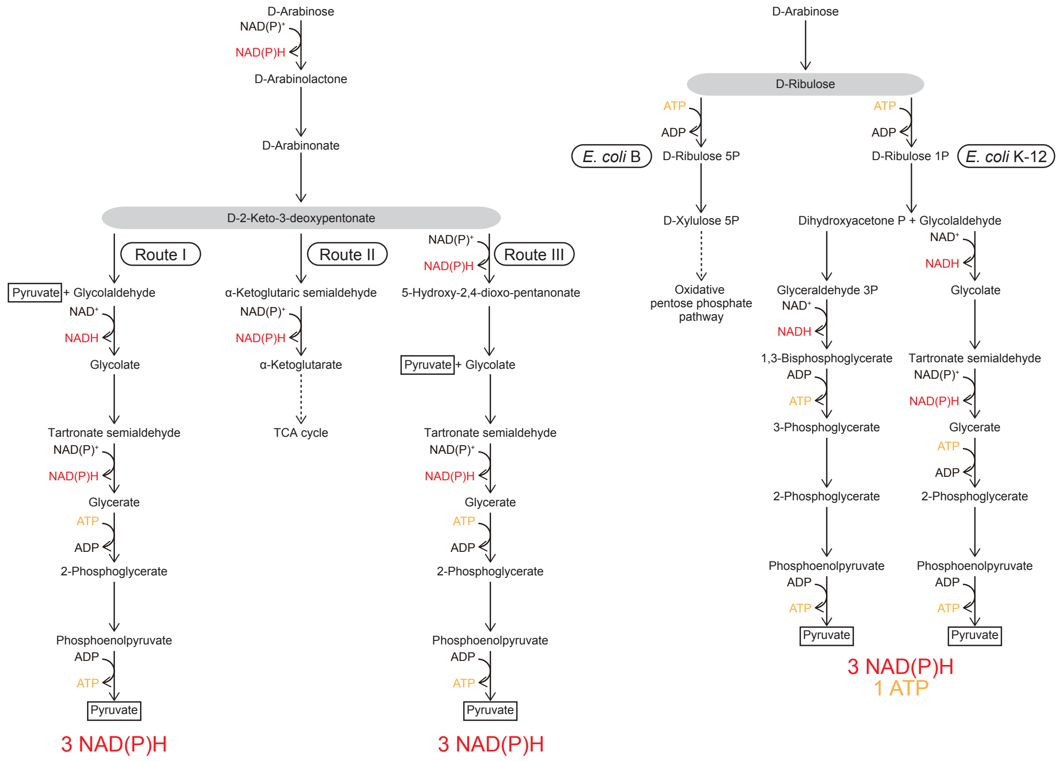

Supplementary Figure S9. Overview of three possible routes for D-arabinose degradation.
